# Conservation of *shh cis*-regulatory architecture of the coelacanth is consistent with its ancestral phylogenetic position

**DOI:** 10.1186/2041-9139-1-11

**Published:** 2010-11-03

**Authors:** Michael Lang, Yavor Hadzhiev, Nicol Siegel, Chris T Amemiya, Carolina Parada, Uwe Strähle, May-Britt Becker, Ferenc Müller, Axel Meyer

**Affiliations:** 1 Department of Biology, University of Konstanz, 78457 Konstanz, Germany; 2 Department of Medical and Molecular Genetics, University of Birmingham, Birmingham B15 2TT, UK; 3 Karlsruhe Institute of Technology, Institute for Toxicology and Genetics, 76021 Karlsruhe, Germany; 4 Developmental Biology Group, Universitat Pompeu Fabra, Parc de Recerca Biomèdica de Barcelona, 08003 Barcelona, Spain; 5 Benaroya Research Institute at Virginia Mason, Seattle, WA 98101, USA; 6 Development and Neurobiology Program, Jacques Monod Institute, 75013 Paris, France; 7 Medical University of Vienna, Medical Genetics, 1090 Vienna, Austria; 8 Center for Craniofacial Molecular Biology, University of Southern California, Los Angeles, CA 90030, USA; 9 Exzellenzcluster CellNetworks, INF 267, 69120 Heidelberg, Germany

## Abstract

**Background:**

The modern coelacanth (*Latimeria*) is the extant taxon of a basal sarcopterygian lineage and sister group to tetrapods. Apart from certain apomorphic traits, its morphology is characterized by a high degree of retention of ancestral vertebrate structures and little morphological change. An insight into the molecular evolution that may explain the unchanged character of *Latimeria *morphology requires the analysis of the expression patterns of developmental regulator genes and their *cis*-regulatory modules (CRMs).

**Results:**

We describe the comparative and functional analysis of the *sonic hedgehog *(*shh*) genomic region of *Latimeria menadoensis*. Several putative enhancers in the *Latimeria shh *locus have been identified by comparisons to sarcopterygian and actinopterygian extant species. Specific sequence conservation with all known actinopterygian enhancer elements has been detected. However, these elements are selectively missing in more recently diverged actinopterygian and sarcopterygian species. The functionality of the putative *Latimeria *enhancers was confirmed by reporter gene expression analysis in transient transgenic zebrafish and chick embryos.

**Conclusions:**

*Latimeria shh *CRMs represent the ancestral set of enhancers that have emerged before the split of lobe-finned and ray-finned fishes. In contrast to lineage-specific losses and differentiations in more derived lineages, *Latimeria shh *enhancers reveal low levels of sequence diversification. High overall sequence conservation of *shh *conserved noncoding elements (CNE) is consistent with the general trend of high levels of conservation of noncoding DNA in the slowly evolving *Latimeria *genome.

## Background

Evolutionary change of *cis*-regulatory regions is not well understood, and there are conflicting observations about how much *cis*-regulatory evolution is linked to diversification of gene expression. Compensatory changes in *cis*-regulatory regions argue for a weak correlation of both [1,2]. Nevertheless, strong DNA sequence conservation of enhancers of developmental regulator genes [3-8] implies purifying selection to keep such regions preserved across species and functionally constrained in their *cis*-regulatory functions. Comparative genomics is widely used for the detection of conserved noncoding elements (CNE) which can be examined experimentally for *cis*-regulatory function [8-12]. Despite certain discussion [13-18], *cis*-regulatory modules (CRM) are regarded as likely targets for adaptive molecular changes that lead to morphological variation [13,16].

The modern coelacanth (*Latimeria*) represents the most basal lineage of living sarcopterygians. Its species diversity is considered to have remained low within its long time of existence of at least 360 million years [19], with a certain peak of species abundance in the Triassic and Jurassic eras. Concomitant with this reduced species divergence, its morphology has remained almost unchanged [20]. *Latimeria *possesses structures such as the intercranial joint that are otherwise known only from long-extinct vertebrates. The vertebral column is poorly developed, and the notochord is retained in adults seemingly serving as the principal axial skeleton [21]. Thus, the few morphological changes may be reflected in its ancestral type genetic makeup. Previous studies are in good agreement with this view. The characterization of the *Latimeria *HOX cluster [22] and procadherin gene cluster [23] provide evidence of the slow evolution of the *Latimeria *genome and conserved noncoding DNA. The orthologous *Otx2 *enhancers FM and AM [24] and the *HoxC8 *early enhancer [25] revealed strong conservation in DNA sequence and in enhancer expression in mouse transgenic experiments.

To study the evolution of the regulatory architecture of a developmental gene, *sonic hedgehog *(*shh*) provides a good candidate. The *shh *gene encodes a morphogen that directs many developmental processes in vertebrates [26-28]. The transcriptional regulation of *shh *is tightly regulated, and its expression in the embryonic midline is specific to the ventral neural tube and the notochord. Those *shh *tissue-specific expression domains are conserved in a wide range of vertebrate species such as in mouse and zebrafish [26,27] but also in the agnathan lamprey [29] and chondrichthyan dogfish [30]. The *cis*-regulatory regions that regulate *shh *expression in the central nervous system and the notochord have been mapped extensively in several species and have been functionally characterized in mouse and zebrafish [31-40]. Thus, *shh *represents an ideal gene locus for a detailed examination of *cis*-regulatory sequence conservation in the context of the slow genome sequence evolution and the ancient type morphology of the coelacanth.

In our report, we demonstrate that the *Latimeria menadoensis shh *locus contains all conserved proximal enhancers shared nonuniformly by fishes and land vertebrates. We provide experimental verification for enhancer activity of the putative *Latimeria *enhancers in transgenic zebrafish and electroporated chick embryos. From DNA sequence comparison of the *shh *locus of different vertebrate lineages, we infer that *Latimeria *conserved noncoding elements represent the ancestral gnathostome set of enhancers that diverged variably during vertebrate evolution.

## Results

### Isolation of the *Latimeria sonic hedgehog *locus

**Figure 1:**
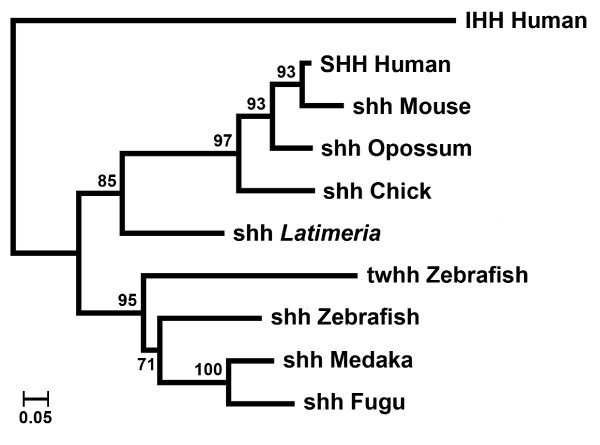
**Phylogeny of vertebrate *shh***. RaxML Maximum likelihood tree [64], (JTT Matrix), with shh protein sequences, rooted with human IHH. Numbers above nodes indicate bootstrap support (100 replicas) [GenBank accession numbers: SHH human: NM_000193.2, *shh *mouse: NM_009170.2, *shh *chick: NM_204821.1, *shh *zebrafish: NM_131063.1, *twhh *zebrafish: NM_131199.2, *shh *Fugu: AY690624.1, IHH human: XM_050846.3]. The deduced cds of Medaka (*Oryzias latipes*) and opossum (*Monodelphis domestica*) were extracted from genome data. The *Latimeria menadoensis shh *cds was deduced from DNA sequence alignments.

Three variants of hedgehog exon 2 [GenBank accession numbers FJ603041, FJ603042 and FJ603043] sequences were obtained by polymerase chain reaction (PCR) from genomic DNA of *Latimeria chalumnae *using degenerate primers. The *shh*-specific paralog was deduced from DNA sequence comparisons and used as a probe to screen a *Latimeria menadoensis *BAC (Bacterial Artificial Chromosome) library [41]. The BAC clone 123-O2 was shotgun sequenced, and approximately 1200 sequence reads resulted in a 5X DNA coverage of the BAC clone. After assembly, a 20-kb contig was obtained that encompasses the *shh *genomic region, spanning from 8 kb upstream to 12 kb downstream of the predicted *shh *start codon [GenBank accession number FJ603040]. Repetitive DNA and interspersed elements made it difficult to align the entire BAC clone sequence in a single contig, and correct assembly was verified for the 20-kb region by PCR amplifications. The *shh *coding portion was deduced from DNA sequence comparisons to mouse and chick *shh*. Phylogeny reconstruction with *shh *coding sequences of higher vertebrate species indicated that indeed the *Latimeria shh *orthologous gene had been sequenced (Figure 1).

**Figure 2:**
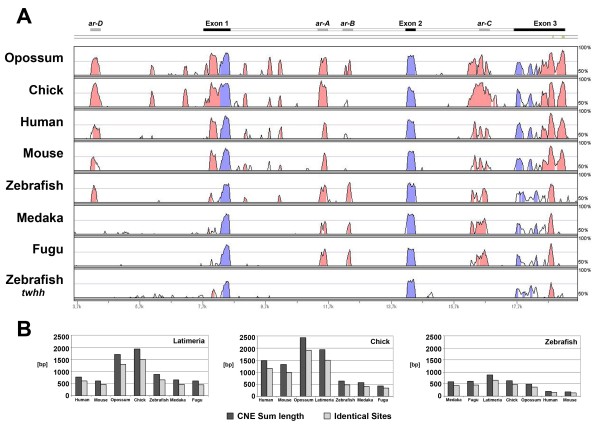
***shh *locus (a) VISTA plot of the *shh *genomic region (*Latimeria menadoensis shh *as reference sequence)**. Shuffle-LAGAN alignment (repeatmasker applied), visualized with mVISTA. Black peaks indicate conservation of coding sequences. The crossbar indicates the deduced *Latimeria shh *gene structure (black: exons). Enhancer locations are indicated by grey bars. Repeats are indicated by yellow bars. **(b) **Overall Conservation of CNE. Histograms present sum lengths of DNA sequence conservation in the *shh *genomic region from pairwise comparisons within the genomic region from SFPE1/*ar-D *enhancer to the *shh *3' end (for zebrafish-, medaka- and fugu from the putative *ar-E *enhancer to the *shh *3' end). Alignments were established with mLAGAN, conservation parameters for CNE identification were set as: Min Y: 50, min Id: 70, Min length: 60. CNE that mapped *shh *untranslated regions were excluded from the analysis.

We identified the homologous conserved sequences in the *Latimeria menadoensis shh *genomic region that were identified previously in various lobe-finned and ray-finned vertebrates [42-45]. Several noncoding conserved sequences were detected in intronic and upstream regions of *shh*, and the distribution and frequency of these conserved blocks followed recognizable patterns. They overlap with the previously characterized enhancer regions of zebrafish and mouse (Figure 2a).

An upstream enhancer, SFPE1, directs *shh *floorplate expression in mouse. Two other enhancers within intron 2, SBE1 and SFPE2, drive *shh *expression in the ventral brain and notochord [32,36]. In zebrafish, the enhancers *ar-A *and *ar-B *in intron 1 and *ar-C *in intron 2 mediate *shh *expression in the notochord and the ventral neural tube. Both *ar-A *and *ar-C *predominantly mediate notochord expression, while *ar-B *predominantly directs *shh *expression in the floorplate [33,40].

The intronic enhancer *ar-A *is well conserved in all analyzed sequences. A second peak of conservation upstream of the *Latimeria shh *gene was detected to correspond to the enhancer *ar-D *in zebrafish or SFPE1 in mouse. Conservation of this region among *Latimeria *and other sarcopterygians was detected in a region of approximately 300 bp, which expanded conservation to zebrafish *ar-D *by 50 bp at the 5- and 150 bp at the 3 end (Additional file 1). Furthermore, the *ar-D *enhancer is not conserved in pufferfishes (e.g., *Takifugu rubripes*) and medaka (*Oryzias latipes*). The third peak of conserved sequence found in *Latimeria *is the region corresponding to *ar-B*, a floorplate-specific enhancer in zebrafish. It showed similarity with actinopterygian and with marsupial species, but with none of the other sarcopterygians for which genomic data are available. This result suggests that the *ar-B *enhancer has been lost independently in the different tetrapod lineages. In opossum, the sequence that retains *ar-B *conservation is interrupted with approximately 230 bp of repetitive DNA. The 5- part of this conservation is further conserved in *shh *loci of placental mammalian species, such as human (Additional file 2). It forms part of a diverged mammalian-specific CNE that vice versa is only rudimentarily conserved in *Latimeria*.

In summary, *Latimeria *has retained conservation of all four putative enhancers that were previously described in the actinopterygian zebrafish. A fourth region with CNE was detected in the second intron. This is the region corresponding to *ar-C *in zebrafish (and SFPE2 enhancer in mouse). This region was previously shown to be strongly conserved in *Latimeria *[34]. Therefore, all four enhancers must have evolved before the split between the lobe-finned and ray-finned fish lineages.

Overall conservation of noncoding DNA was estimated to get a more quantitative measure of *shh *CNE in the different lineages. We calculated the sum lengths of CNE from pairwise comparisons of two *shh *genomic regions, spanning from the SFPE1/*ar-D *upstream enhancer to the *shh *3'-UTR (Figure 2b). As no conservation was found to *ar-D *in medaka and fugu, for further analysis, the 5' limit of teleost *shh *genomic regions was defined to be a teleost-specific CNE further upstream (Additional file 3). Notably, *Latimeria *shows the highest sequence similarity with chick among vertebrates. Conservation is comparatively high with opossum but remarkably reduced when compared to mouse and human. Compared to actinopterygian species, sarcopterygian *shh *genomic regions share more CNE relative to their phylogenetic distance [46-48].

Conserved *Latimeria *enhancer sequences were analyzed for putative transcription factor binding sites using the JASPAR CORE vertebrata database [49] (Additional file 1). Binding motifs of those transcription factors were searched, which are known to be expressed in the neural tube, especially the floorplate [36,50]. Several elements were found, such as homeobox or IRX elements, and we detected putative FoxA2 binding sites in the *ar-A *and *ar-D *enhancer. The *ar-D *FoxA2 putative binding site was only conserved among sarcopterygians but not in zebrafish. FoxA2 is a floorplate-specific transcription factor, and a FoxA2 element was also shown to be functional in the mouse SFPE2 enhancer [36].

Finally, we examined the rates of divergence within conserved *shh *enhancer sequences among sarcopterygian species with the relative rate test [51]. This test estimates different rates of DNA sequence diversification between two lineages by comparing them to an outgroup sequence. The DNA sequence alignments were obtained from partial enhancer regions that were found in all analyzed species (Table 1, Additional file 4). We used either *Latimeria *or zebrafish as outgroup sequences. Congruent to the overall conservation, the mammalian enhancer sequences showed elevated rates of divergence compared to chick or *Latimeria*, with opossum putative enhancer sequences at intermediate rates and the placental mammalian species at highest rates (Table 1). In conclusion, all CNEs that were previously identified either in mouse or in zebrafish are present in *Latimeria *and thus are candidate enhancers of *shh *expression in the *Latimeria *embryonic midline.

**Table 1:** Relative rate tests with *shh *partial enhancer sequences.*

*ar-D*/SFPE1						
Ingroup		Outgroup	Identical Sites	Divergent Sites	**χ**^**2 **^**(1° freedom)**	Probability
Mouse	Chick	Lme	122	8	27.52	0.00000
Human	Chck	Lme	126	1	17.79	0.00002
Opo	Chick	Lme	144	1	9.97	0.00159
Lme	Chick	Danio	129	9	1.33	0.24821
***ar-A***						
**Ingroup**		**Outgroup**	**Identical Sites**	**Divergent Sites**	**χ^2 ^(1° freedom)**	**Probability**

Mouse	Chick	Lme	98	9	8.17	0.00427
Human	Chick	Lme	98	4	12.80	0.00035
Opo	Chick	Lme	105	2	3.56	0.05935
Lme	Chick	Danio	103	5	2.00	0.15730
***ar-C***						
**Ingroup**		**Outgroup**	**Identical Sites**	**Divergent Sites**	**χ^2 ^(1° freedom)**	**Probability**

Mouse	Chick	Lme	65	1	13.50	0.00024
Human	Chick	Lme	72	1	9.31	0.00228
Opo	Chick	Lme	73	2	6.25	0.01242
Lme	Chick	Dano	62	3	1.60	0.20590

*The equality of evolutionary rate between ingroup sequences is tested using the outgroup sequence. The χ^2 ^test statistic and probability values are listed. Enhancer alignments are shown in Additional file 4. Species generic name abbreviation Lme: *Latimeria menadoensis*, Danio: *Danio rerio *(zebrafish), Opo: *Monodelphis domestica *(opossum).

### Functional mapping of orthologous enhancers

The enhancers *ar-A*, *ar-B *and *ar-C *of zebrafish and the SBE1, SFPE1 and SFPE2 of mouse *shh *were mapped by functional analysis to overlap exactly with conserved noncoding sequences [32-34,36,40,43,44]. However, the SFPE1 orthologous enhancer in zebrafish, denoted as *ar-D*, is a 2.4-kb fragment that is too large to suggest that the CNE within contains the functional enhancer. The fine mapping of the zebrafish *ar-D *enhancer was carried out to confirm that the conserved zebrafish *ar-D *sequence is reponsible for the enhancer effect in the 2.4-kb fragment. This would improve the predictive value of this CNE in predicting a *Latimeria *enhancer. Five partially overlapping DNA fragments of 500 bp or 700 bp were coinjected with a minimal 0.8-kb *shh *promoter construct [31] linked to a *LacZ *reporter (Additional file 5). Weak activation of notochord and floorplate expression was observed for fragments that represent the *shh *2.5- to 1.5-kb upstream region (fragments 2-5). The expression was not considerably higher than background staining and was probably originated from the 0.8-kb proximal *shh *promoter. Specific reporter gene expression in the anterior floorplate (anterior to the level of the yolk extension) was observed for DNA fragment 1, ranging from position -836 to -1339. The region that is responsible for the *ar-D *enhancer effect overlaps fully with a CNE that is present in all the other compared sarcopterygian sequences (Figure 2a). This region is further part of the mouse SFPE-1 enhancer. We thus postulated that this CNE would also function as a specific enhancer in *Latimeria*.

**Figure 3:**
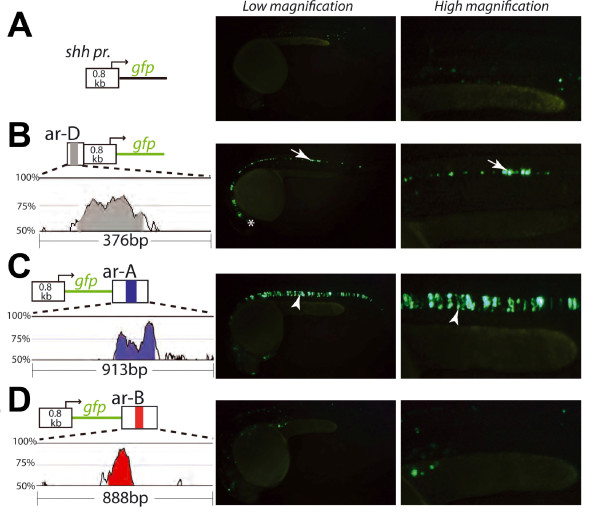
**Functional assay of *Latimeria ar-A*, *ar-B*, and *ar-D *enhancers in zebrafish**. Two fluorescent images are shown for each embryo. Low magnification displays the whole embryo and high magnification focuses on the trunk above the yolk extension. Schematic representations of the injected zebrafish (z) and *Latimeria *(l) promoter (pr) and enhancer reporter constructs are shown on the left side of each panel **(a-d) **VISTA plot comparisons of the zebrafish and *Latimeria *enhancer regions are shown below reporter constructs and indicate the degree of conservation. Conservation identity greater than 70% is highlighted in color. **(a) **Embryo injected with control construct containing the 0.8 kb (form the transcriptional start site) zebrafish *shh *promoter, linked to GFP. **(b-d) **Embryos injected with reporter constructs containing the minimal zebrafish *shh *promoter and one of the *Latimeria shh *enhancers *ar-D *(b) *ar-A *(c) and *ar-B *(d). GFP expression in the floorplate is indicated by arrows and the arrowheads point at expression in the notochord.

To check whether the *Latimeria *conserved noncoding sequences had enhancer activity, reporter gene expression analysis was conducted in transient transgenic zebrafish embryos. This analysis had already been carried out with *Latimeria ar-C *in a previous study [34], and the aim here was to analyze other conserved regulatory regions that potentially drive *shh *midline expression. Conserved noncoding elements of *Latimeria shh *intron 1 located between exon 1 and the intronic enhancer *ar-A *did not drive specific reporter gene expression (data not shown). *Latimeria *putative enhancer orthologs *ar-D*, *ar-A *and *ar-B *were cloned into the above-mentioned *shh *minimal promoter constructs, containing GFP (Green Fluorescent Protein) instead of a LacZ reporter. Transient mosaic expression of GFP was measured as read out of reporter construct activity 24 hours after injection of zebrafish zygotes (Figure 3). The reporter expression directed by the zebrafish *shh *upstream region resembled the tissue-specific expression of the isolated *ar-D *enhancer (Additional file 5). GFP expression was observed in the ventral brain and in the anterior parts of the floorplate (data not shown) [33,40]. Similarly, *Latimeria ar-D *also directed floorplate-specific GFP expression (Figure 3b). However, the reporter expression was extended to the posterior parts of the floorplate. Among GFP-expressing embryos, fluorescence in the posterior floorplate cells (posterior to the start of the yolk extension) was detected in only 4% of specimens with the zebrafish upstream region but in 75% of corresponding embryos with *Latimeria ar-D *(Table 2). These results indicate that the CNE in the *Latimeria *upstream region is a functional midline enhancer which has similar but not identical activity in zebrafish to the zebrafish *ar-D *enhancer. On the basis of our DNA sequence comparison, *Latimeria ar-D *was found to contain sarcopterygian specific sequences with a puative FoxA2 element (92% match). Potentially, these elements can account for the posteriorly extended expression direction of *Latimeria ar-D *in the ventral neural tube of zebrafish.

**Table 2:** Classification of GFP-expressing Embryos from Transient expression Experiments.

Reporter construct	Floor plate		Notochord		Expressing embryos	Injected embryos	Freq.
	<15 cells	>15 cells	<15 cells	>15 cells	N°	N°	%
*0.8shh:gfp*	0	0	0	0	22	102	22
*2.4shh:gfp*	A: 0 P: 164	A: 171 P: 7	0	0	171	234	73
*lar-D:0.8shh:gfp*	A: 0 P: 46	A: 182 P: 136	0	0	182	268	68
*0.8:shh:gfp:lar-A*	0	0	0	123	123	167	74
*0.8shh:gfp:lar-B*	3	0	0	0	31	143	22

N° GFP-positive cells per embryo of each construct in the expression domains of interest. The abundance of GFP-expressing embryos below and above the threshold is shown (15 GFP-positive cells per embryo). Abbreviations: A: anterior floor plate; P: posterior floor plate.

The *Latimeria *conserved sequence that overlaps with the zebrafish *ar-A *enhancer drives specific expression in the notochord indistinguishable to the activity of zebrafish *ar-A *(Figure 3c). This result indicates that *Latimeria *indeed carries a functional *ar-A *enhancer. However, no activity could be detected from the *Latimeria ar-B *orthologous fragment when the conserved region alone or a broader region of 888 bp were tested at 24 hpf and 48 hpf (hours post fertilization) (Figure 3d and data not shown). Since a *Latimeria ar-B *enhancer may have diverged from that in zebrafish, a sarcopterygian model system was chosen to test putative *Latimeria *enhancer function. *Latimeria ar-B *enhancer activity was tested in chick, which is evolutionarily more closely related to *Latimeria *than zebrafish and shows higher overall noncoding DNA sequence similarity with *Latimeria*. This might also imply higher conservation of *cis*- and *trans*-interactions. Besides, the *ar-D *enhancer was also tested for reporter expression activity in chick. Since *Latimeria ar-D *directed posterior floorplate expression in zebrafish, the question arose whether this anteroposterior restricted pattern of activity is due to a zebrafish-specific *trans-*effect or if it is a more general response also seen in chick. As many transcription factors are supposed to have pleiotropic functions [13], the *trans*-acting factors may thus evolve independently to responsive *cis*-regulatory regions. In this case, *trans*-factors may be present in the chick neural tube, although the *ar-B *enhancer itself is absent in that species.

**Figure 4:**
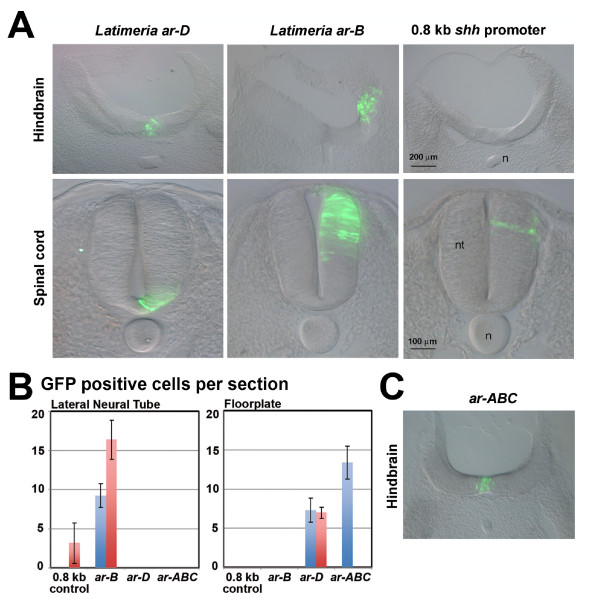
**Functional assay of *Latimeria ar-B*, and *ar-D *enhancers in chick**. **(a) **GFP expression in the hindbrain and in the posterior spinal cord obtained with the *Latimeria *enhancer constructs and with the zebrafish 0.8 kb *shh *promoter region as negative control. GFP expression was detected by immunostainings, except for *ar-D *expression in hindbrain where the direct GFP fluorescence is shown. The neural tube (nt) and the notochord (n) are indicated in the images. Sections of the hindbrain are posterior to the tegmentum (posterior to midbrain-hindbrain boundary and anterior to the otic vesicles). Sections of the spinal cord are at the level of somite 33. **(b) **Histograms, presenting amounts of GFP posivite cells in the floorplate and lateral neural tube. The number of fluorescent cells were counted from prepared sections of electroporated embryos. **(c) **Positive control electroporation with the construct -2.4shh:gfpABC [33].

The previously described *Latimeria ar-B *and *ar-D gfp-*reporter constructs were electroporated either in the hindbrain or in the spinal cord of chick embryos. GFP reporter gene expression was compared to control electroporations with the zebrafish 0.8-kb *shh *promoter construct, carried out in parallel. We also carried out another control experiment with the construct -2.4shh:gfpABC [33]. This plasmid contains regulatory regions equivalent to plasmid I1+I2-2.2shh::lacZ characterized by Müller *et al*. (2000) [52] and was shown to be specifically expressed in the floorplate of the chick neural tube (Figure 4, Table 3). Weak or no background GFP expression in the spinal cord was detected with the 0.8-kb *shh *control plasmid. Electroporation with the *Latimeria ar-B *enhancer resulted in significantly enhanced GFP expression. However, the expression pattern was not specific to the ventral neural tube. This result suggests that the *Latimeria ar-B *sequences carry enhancer activity but are either not restricted to the ventral neural tube or are interpreted in a nonspecific fashion in chick. The *Latimeria-arD *enhancer directed tissue-specific GFP expression in the floorplate, both in hindbrain and in the posterior spinal cord. This result is similar to those observed in transient transgenic zebrafish (Figure 3b).

**Table 3:** Number of GFP-expressing chick embryos in eletroporation experiments. N° GFP-positive embryos/N° electroporated embryos.

Reporter construct	Hindbrain (n)	Spinal cord (n)
*-2.4shh:gfpABC*	^7^/_7_	--
*0.8shh:gfp*	^0^/_4_	^1^/_4_
*0.8shh:gfp:lar-B*	^6^/_8_	^4^/_4_
*lar-D:0.8shh:gfp*	^5^/_7_	^2^/_3_

Summarizing, only the predicted *Latimeria *conserved enhancers *ar-D *and *ar-A *were found to be functional in zebrafish. The *Latimeria ar-B *enhancer was providing unspecific enhancement of GFP expression in chick, both in the hindbrain and in the posterior neural tube. These results suggest that all three regions are functional enhancers in *Latimeria*, albeit that experimental verification in *Latimeria *is currently not feasible.

## Discussion

### Conservation of *Latimeria shh *noncoding DNA

The *shh *genomic region of *Latimeria menadoensis *reveals conservation of all four actinopterygian *shh *midline enhancers, which indicates an ancestral-like and rather unchanged *cis*-regulatory architecture of *Latimeria shh*. Several previous studies with *Latimeria *enhancers of different loci or genomic regions are in agreement with this interpretation. The analysis of the *Latimeria *procadherin gene clusters [23] described the *Latimeria *genome as being very stable and having suffered very little from diversifications such as gene duplications or gene conversion events. The characterization of *Latimeria *HOX clusters revealed a consistently slower diversification of *Latimeria *CNE with repsect to tetrapods [22]. The *Latimeria *orthologous *Otx2 *enhancers FM and AM [24] as well as the *HoxC8 *early enhancer [25] revealed strong DNA sequence conservation across vertebrates. *Otx2 *enhancer expression direction was similar to that obtained with skate orthologs, and the *HoxC8 *early enhancer was described to direct gene expression in mouse, similar to the mouse ortholog. Our study extends the observations cited above to the characterization of a set of proximal regulatory modules within a locus. With availability of the *Latimeria *genome sequence, a more comprehensive comparative genomic analysis should be possible with a multitude of developmental regulator genes. Such an approach may also reveal how far conservation of *cis*-regulatory regions at developmental regulator genes is related to its ancient type morphology.

**Figure 5:**
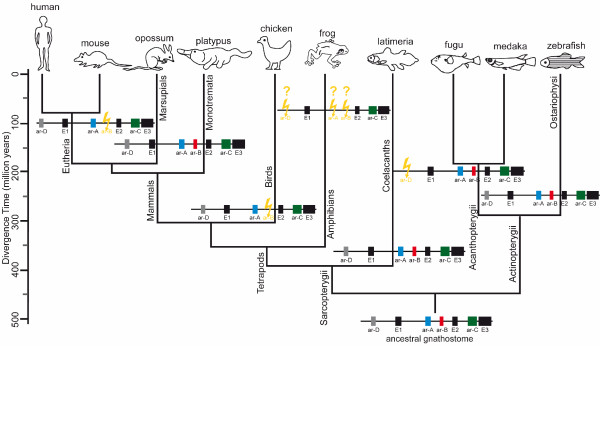
**Hypothetical evolution of enhancer structure of *shh *loci in vertebrates**. The structure of the *shh *locus is schematically represented for each vertebrate linage on the tree showing vertebrate evolutionary relationships. The enhancers are highlighted in color: blue: *ar-A*; red: *ar-B*; green: *ar-C *and gray: *ar-D*; exons (E1, E2, E3) in black. The lightning symbol (yellow) indicates enhancer loss in the corresponding vertebrate linage. Question marks indicate incomplete sequence data. The species divergence time scale was adapted from literature data [46-48].

Conservation of the four enhancers *ar-A*, *ar-B*, *ar-C *and *ar-D *in *Latimeria *and zebrafish reveals preservation of an ancestral set of enhancers that originated before the split between ray-finned and lobe-finned vertebrates (Figure 5). Lineage-specific losses of *ar-D *and *ar-B *are observed in more derived species. Those are likely structural enhancer rearrangements that display a functional turnover within a cooperating system [33] of *cis*-regulatory regions at the *shh *locus.

So far, there is not enough data available on *shh *proximal genomic regions of ancestral gnathostomes (represented by chondrichthyes such as sharks). However, there is reason to speculate that the intronic and upstream enhancers are potentially present in such species. Embryonic expression of *shh *in the embryonic midline has been shown to be strongly conserved in distantly related vertebrate species such as lamprey [29] or dogfish [30].

The analysis of total conservation of CNE (Figure 2b) in the *shh *orthologous genomic regions indicates interesting trends in the conservation of CNE across actinopterygian and sarcopterygian species. A lower overall conservation is observed among teleost species. Less conservation of noncoding DNA in teleost lineages has also been reported previously in DNA sequence comparisons of vertebrate Hox clusters [53], which was related to the fish-specific genome duplication [54]. Among sarcopterygians, we found an elevated similarity of CNE between *Latimeria *and chick and lower conservation to the mammalian sequences. It indicates that the *shh *genomic region has stayed remotely constrained across sarcopterygian evolution but has more recently diversified in the mammalian lineage. Comparisons of the conserved blocks of the enhancers *ar-A*, *ar-C *and SFPE1/*ar-D *by relative rate tests [51] are in line with this interpretation. Mammalian orthologs show substantially more diversification than chick or *Latimeria*. Likewise, the mouse *ar-C *orthologous enhancer SFPE2 is floorplate-specific and has been reported to be inactive in transgenic zebrafish [34]. Chick and *Latimeria *orthologs [34] showed notochord specific expression, similar to the zebrafish ortholog [32,34,40].

### Expression specificity of *Latimeria *and zebrafish orthologous enhancers

The analysis of expression of putative *Latimeria *orthologous enhancers in transgenic zebrafish and chick provided confirmation of functionality, though it cannot definitively corroborate the endogenous tissue specificity in *Latimeria*. Although similar, there are qualitative differences in expression patterns generated by the *Latimeria ar-D *sequence and the 2.4-kb zebrafish *shh *upstream region that contained the *ar-D *enhancer. This region directed expression in anterior parts of the floorplate and the ventral brain. The expression of the *Latimeria ar-D *enhancer in the posterior floorplate can potentially result from enhancer regions that are only conserved among sarcopterygians (Additional file 1). Those regions can contain elements, such as the the putative FoxA2 binding site, which are bound by floorplate-specific transcription factors. A FoxA2 binding site was previously shown to be functional in the mouse floorplate-specific enhancer SFPE2 [36] and the mouse *ar-D *orthologous enhancer (SFPE1), characterized in mouse, also revealed to drive reporter gene expression in posterior parts of the floorplate [32]. However, as these regions were analyzed in different model organisms, these data may not fully be comparable. Also, the mouse SFPE2 enhancer is inactive in zebrafish [34] but contains a functional FoxA2 element. Thus, other elements may be responsible or additionally required for posterior floorplate expression of *Latimeria ar-D*. The subtle differences in reporter gene expression by orthologous enhancers from different species have also been reported previously and appear to be a common phenomenon [24,33,34,36,55]. Divergent expression direction may possibly be explained by different binding site compositions of the orthologous enhancers or differing transcription factor availability and affinity to the same set of transcription factor binding sites among different species.

Similar to the observed changes in *ar-D *activity, *Latimeria ar-B *proved also to be differently interpreted in zebrafish, compared to its zebrafish homolog. In the transgenic reporter assays employed, *Latimeria ar-B *did not drive reporter gene expression in zebrafish. However, an unspecific activation of GFP expression by *Latimeria ar-B *was observed in chick embryos. This suggests that *ar-B *is a functional *cis*-regulatory region in the first place. The lack of tissue-specific expression might simply reflect different interpretations of *ar-B *enhancer elements in zebrafish, chick and *Latimeria*. We observed conserved sequences among teleost species that map zebrafish *ar-B *but that are not present in *Latimeria *(Additional file 1). Those regions may be required to obtain specific reporter gene expression. Again, changes in transcription factor binding site composition and transcription factor availability and affinity can result in reduced responsiveness of the reporter system applied in different species. Alternatively, *Latimeria ar-B *might require the cooperation with other enhancers for tissue-specific activity. As zebrafish *shh *floorplate enhancers cooperate with each other [33], there might be a balancing gain and loss of enhancer functions among the complete set of *shh *midline enhancers that together preserve the tissue-specific activity. Similarly, *shh *and *twhh *reporter constructs showed ectopic activity when a limited number of enhancers were assayed out of context of additional enhancers [33,56]. Following this speculation, the loss of tissue-specific activity of one enhancer might be complemented by a gain of tissue specificity through synergism with another enhancer. Keeping in mind the limitations, cross-species reporter analysis remains the only approach to obtain expression data of *Latimeria cis-*regulatory elements.

An open question that has not been addressed in this study is whether *Latimeria *carries orthologous putative *shh *long-range enhancers. A CRM that regulates *shh *limb bud expression is located 1 mb away from mouse *shh *within the lmbr1 locus. This enhancer was found to be conserved in most vertebrate lineages, including chondricthyes [35,38,39,57-60]. Other *shh *long-range enhancers have been detected, such as endoderm-specific enhancer elements [58] and the ventral forebrain specific enhancer SBE2. This latter CRM is conserved only in sarcopterygians and is located in humans at a distance of 460 kb upstream of SHH [35,37]. A *Latimeria *genome project will therefore be immensely useful in addressing the presence such long distance *cis*-regulatory modules.

## Conclusions

The *Latimeria menadoensis shh *genomic region represents a locus with the ancestral set of enhancers that emerged before the split of lobe-finned and ray-finned fishes. In contrast to more derived vertebrate lineages that exhibit losses and rearrangements of *shh *enhancers, *Latimeria *reveals low levels of enhancer sequence evolution and high overall conservation of noncoding DNA at the *shh *proximal genomic region. The conserved *cis*-regulatory architecture of this set of *shh *midline enhancer is consistent with slow rates of evolution of the *Latimeria *genome. The high overall sequence conservation of *shh *CNE may be indicative of a high retention of ancestral *cis*-regulatory DNA in the *Latimeria *genome.

## Methods

### Amplification of an ExonII specific probe of *Latimeria menadoensis*

The DNA sequence of *shh *Exon II was amplified by PCR from 100 ng of genomic DNA of *Latimeria chalumnae *using the oligonucleotides SHHE2F1: CNATHTCNGTRATGAACCAGTGG and SHHE2R1: CTGCTTTSACNGARCARTGDAT. The amplification product was ligated into the PCRII vector (Invitrogen, Carlsbad, CA, USA), transformed, and single clones from transformations were isolated and prepared. The insert of each clone was sequenced using the primers M13(-20) and M13(reverse) on an ABI PRISM 3100 Genetic Analyzer (Applied Biosystems, Foster City, CA, USA), using the Big Dye Termination Reaction chemistry (Applied Biosystems). Suitable clone inserts were used as DNA template for the preparation of a 233-bp *shh*-specific ^32^P-labeled probe for BAC library screening. ^32^P-labeling was achieved by random priming [61].

### BAC library Screening and BAC-clone sequencing

The *Latimaria menadoensis *BAC library VMRC4 [41] was screened for *shh*-specific BAC clones. One nylon filter set was hybridized overnight in 0.6 M NaCl, 0.02 M ethylenediaminetetraacetic acid (EDTA), 0.2 M Tris pH 8.0, 0.5% sodium dodecyl sulfate (SDS) and 0.05% sodium pyrophosphate at 65°C with the *shh*-specific probe. The filters were washed twice with 1 × saline-sodium citrate (SSC), 0.1% SDS, once for 30 minutes at room temperature and once for 40 minutes at 37°C. Three *shh*-positive BAC clones were isolated.

Large-scale BAC clone preparation was obtained with the Large Construct Kit (Qiagen, Hilden, Germany). A total of 20 μg of BAC clone DNA in 500 μl TE (10 mM Tris, 1 mM EDTA), pH 8.0, was fragmented by sonication (Branson Sonifier, Danbury, CT, USA) with 4 × 1-second pulses at 300 W (5-mm microtip). The 2- to 3-kb fraction was isolated from the sheared DNA by preparative gel electrophoresis. DNA fragments were end-polished with Klenow enzyme (Roche, Basel, Switzerland) and blunt-end ligated into pUC18 vector (Roche). Resulting bacterial clones from transformations were isolated into 384-well plates and grown in 1 ml LB (Luria-Bertani) broth (50 μg/ml ampicillin).

The subcloned BAC clone 123-O_2 _was sequenced using the shotgun approach. Clone plasmid DNA was prepared manually. The inserts were sequenced directly with the universal primers M13(-20) and M13(reverse) as mentioned above. Sequences were quality trimmed with PHRED [62] and assembled with the Sequencher software (Gene Codes, Ann Arbor, MI, USA). DNA contigs were aligned to vertebrate *shh *sequences and to conserved noncoding elements of *shh *genomic regions, obtained from the UCSC genome browser [63]. Contig gaps were closed by PCR with gap-specific primers and direct sequencing. Phylogeny reconstruction with *shh *coding sequences was performed with RAxML [64].

### Analysis of conserved noncoding sequences

The *shh *genomic regions of different vertebrate species were extracted with the UCSC genome browser or Ensembl genome browser (human SHH: hg18_dna range=chr7:155271852-155314196, mouse *shh*: mm8_dna range=chr5:28769694-28815809, opossum *shh*: monDom4_dna range=chr8:217895000_217995000 chick *shh*: galGal3_dna range=chr2: 8007268-80525245, zebrafish *shh*: danRer4dna range=chr7_49518680-49540684, zebrafish *twhh*: danRer4_dna range=chr2: 27256710-27288019, medaka *shh*: oryLat2_dna range=chr20: 17728012-17784191, Fugu *shh*: combination of two sequences: AJ507296.1 and fr1_dna range=chrUn:293195538-293226682 (misassembly in the Fugu genome sequence around shh exon1)). Annotations of genomic regions were done manually by DNA sequence comparisons and repetitive DNA was masked with repeatmasker [65] using wublast, default speed/sensitivity, masking interspersed and simple repeats. Genomic regions were aligned with the program Shuffle-LAGAN and alignments were visualized with the program VISTA (mVISTA, [66]; LAGAN Alignment program, [67]. Conserved sequence blocks were also extracted using VISTA.

For relative rate tests, alignment blocks were determined as conserved sequences that are found in all compared DNA sequences. Alignment blocks were established with Dialign TX [68], and relative rate tests were performed with MEGA4 [69].

### Subcloning of enhancers and transgenic analysis

*Latimeria shh *enhancers were amplified by PCR from BAC clone DNA using specific oligonucleotides (Additional file 6). The oligonucleotide pairs optionally contained the restriction sites *Sal*I/*Pst*I, *Not*I/*Sac*II or *Not*I/*Kpn*I. PCR products were cloned into linearized plasmid vectors 0.8-kb shh:GFP [31,34]. Microinjection solution (10-20 ng/μl circular plasmid DNA and 0.1% phenol red) was injected through the chorion into the cytoplasm of zebrafish zygotes. Transient mosaic expression of GFP in 24-hours old embryos was analyzed using a fluorescent stereomicroscope (Leica MZ FLIII, Leica Microsystems, Heerbrugg, Switzerland).

The zebrafish *ar-D *enhancer and the *Latimeria ar-G *fragment was characterized in coinjection experiments, essentially as described in Müller *et al*. (1999) [40]. Analysis of 24 hours old embryos was then carried out as described by Chang *et al*. (1997) [31]. Transient mosaic expression was measured as readout of reporter construct activity by counting β-Gal-stained cells. PCR-amplified fragments of zebrafish *shh *upstream DNA regions were obtained with specific oligonucleotides (Additional file 6).

*In vivo *experiments with chick embryos were performed by *in ovo *electroporation. Eggs from White-Leghorn chickens were incubated at 38.5°C in an atmosphere of 70% humidity. Embryos were staged following Hamburger and Hamilton (HH) [70]. Chick embryos were electroporated with 2 mg/ml supercoiled plasmid DNA with 50 ng/ml Fast Green. Electroporations were carried out as performed by Müller *et al*. (2000) [52]. This method was previously shown to yield transfections of dorsal, lateral and ventral cells of the spinal cord with transfection bias in lateral regions [52]. Briefly, DNA was injected into the dorsal lumen of HH stage 11-12 neural tubes at two different levels: hindbrain or spinal cord. Electrodes were placed at both lateral sides encompassing the dorsal and ventral extremes of the neural tube. Electroporation was carried out in one direction (shown on the right side of Figure 4) so that the nonelectroporated half of the neural tube served as a negative control. Electroporation was performed with the Edit Type Cuy21 (Nepa Gene, Ichikawa, Chiba, Japan) electroporator delivering eight 50-ms square pulses of 10 V. Transfected embryos were allowed to develop for 24 hours.

Chick embryos were fixed for 4 hours at 4°C in 4% paraformaldehyde in PBS, rinsed, embedded in 5% agarose, 10% sucrose and sectioned in a Leica vibratome (VT 1000S, Leica Microsystems). Immunostaining was performed following standard procedures. Briefly, sections were blocked with 10% goat serum in PBS-T (1x PBS (137 mM NaCl, 2.7 mM KCl, 10 mM Na_2_HPO_4_, 2 mM KH_2_PO_4_, pH 7.4), 0.1% Tween-20), incubated overnight with a rabbit anti-GFP antibody (Molecular Probes, Invitrogen) and detected using an anti-rabbit Alexa 488-conjugated secondary antibody (Molecular Probes). Images were collected by fluorescence microscopy.

## List of Abbreviations used

(CRMs): *cis*-regulatory modules; (CDS): coding sequence; (CNE): conserved non-coding elements.

## Competing interests

The authors declare that they have no competing interests.

## Authors' contributions

ML, AM, FM and YH designed the experiments. ML, NS and MBB sequenced the BAC clone and analyzed the data. CTA isolated the *Latimeria shh *containing BAC clones. YH prepared the *Latimeria shh *enhancer constructs, and YH and CP performed the *Latimeria *enhancer transgenic analysis. US and FM provided zebrafish enhancer coinjection analysis. ML, FM, CTA, YH and AM wrote the manuscript.

## Supplementary Material

Additional file 1 **DNA sequence alignments**. Alignments of *shh *enhancers *ar-A*, *ar-B*, *ar-C*, and *ar-D *with putative transcription factor binding sites. Click here for file

Additional file 2 ***ar-B *enhancer conservation**. VISTA plot of the *ar-B *specific genomic region. Shuffle-LAGAN alignment, visualized with mVISTA. Click here for file

Additional file 3 **VISTA plot of the *shh *genomic region**. VISTA plot with zebrafish *shh *as reference sequence. Shuffle-LAGAN alignment, visualized with mVISTA. This figure is used for indication of the putative *ar-E *CNE that is found in zebrafish, medaka and fugu. Click here for file

Additional file 4 **enhancer blocks**. Conserved enhancer sequences used for Relative Rate Tests are presented. Click here for file

Additional file 5 **Mapping of zebrafish *ar-D***. The experimental identification of the zebrafish *ar-D *enhancer is summarized. Click here for file

Additional file 6 **Oligonucleotides**. The oligonucleotides for sub-cloning of enhancers and transgenic analysis are listed. Click here for file

## References

[B1] Ludwig MZ Functional evolution of noncoding DNA Curr Opin Genet Dev 2002 12 634 639 10.1016/S0959-437X(02)00355-6 12433575

[B2] Ludwig MZ Palsson A Alekseeva E Bergman CM Nathan J Kreitman M Functional evolution of a *cis*-regulatory module PLoS Biol 2005 3 e93 10.1371/journal.pbio.0030093 15757364 PMC1064851

[B3] Bejerano G Pheasant M Makunin I Stephen S Kent WJ Mattick JS Haussler D Ultraconserved elements in the human genome Science 2004 304 1321 1325 10.1126/science.1098119 15131266

[B4] Plessy C Dickmeis T Chalmel F Strahle U Enhancer sequence conservation between vertebrates is favoured in developmental regulator genes Trends Genet 2005 21 207 210 10.1016/j.tig.2005.02.006 15797614

[B5] Sandelin A Bailey P Bruce S Engstrom P Klos J Wasserman W Ericson J Lenhard B Arrays of ultraconserved non-coding regions span the loci of key developmental genes in vertebrate genomes BMC Genomics 2004 5 99 10.1186/1471-2164-5-99 15613238 PMC544600

[B6] Vavouri T Walter K Gilks W Lehner B Elgar G Parallel evolution of conserved non-coding elements that target a common set of developmental regulatory genes from worms to humans Genome Biol 2007 8 R15 10.1186/gb-2007-8-2-r15 17274809 PMC1852409

[B7] Venkatesh B Kirkness EF Loh YH Halpern AL Lee AP Johnson J Dandona N Viswanathan LD Tay A Venter JC Strausberg RL Brenner S Ancient noncoding elements conserved in the human genome Science 2006 314 1892 10.1126/science.1130708 17185593

[B8] Woolfe A Goodson M Goode D Snell P McEwen G Vavouri T Smith S North P Callaway H Kelly K Highly conserved non-coding sequences are associated with vertebrate development PLoS Biol 2005 3 e7 10.1371/journal.pbio.0030007 15630479 PMC526512

[B9] Amemiya CT Gomez-Chiarri M Comparative genomics in vertebrate evolution and development J Exp Zool A Comp Exp Biol 2006 305A 672 682 10.1002/jez.a.308 16902957

[B10] Boffelli D Nobrega M Rubin E Comparative genomics at the vertebrate extremes Nat Rev Genet 2004 5 456 465 10.1038/nrg1350 15153998

[B11] Hardison RC Conserved noncoding sequences are reliable guides to regulatory elements Trends Genet 2000 16 369 372 10.1016/S0168-9525(00)02081-3 10973062

[B12] Pennacchio LA Rubin EM Genomic strategies to indentify mammalian regulatory sequences Nat Rev Genet 2001 2 100 109 10.1038/35052548 11253049

[B13] Carroll SB Evo-devo and an expanding evolutionary synthesis: a genetic theory of morphological evolution Cell 2008 134 25 36 10.1016/j.cell.2008.06.030 18614008

[B14] Hoekstra HE Coyne JA The locus of evolution: evo devo and the genetics of adaptation Evolution 2007 61 995 1016 10.1111/j.1558-5646.2007.00105.x 17492956

[B15] Lynch VJ Wagner GP Resurrecting the role of transcription factor change in developmental evolution Evolution 2008 62 2131 2154 10.1111/j.1558-5646.2008.00440.x 18564379

[B16] Prud'homme B Gompel N Carroll SB Emerging principles of regulatory evolution Proc Natl Acad Sci USA 2007 104 8605 8612 10.1073/pnas.0700488104 17494759 PMC1876436

[B17] Wagner GP Lynch VJ The gene regulatory logic of transcription factor evolution Trends Ecol Evol 2008 23 377 385 10.1016/j.tree.2008.03.006 18501470

[B18] Wray GA The evolutionary significance of *cis*-regulatory mutations Nat Rev Genet 2007 8 206 216 10.1038/nrg2063 17304246

[B19] Cloutier R Forey PL Diversity of extinct and living actinistian fishes (*Sarcopterygii*) Env Biol Fishes 1991 32 59 74 10.1007/BF00007445

[B20] Cloutier R Patterns, trends, and rates of evolution within the actinistia Env Biol Fishes 1991 32 23 58 10.1007/BF00007444

[B21] Griffith RW Mathews MB Umminger BL Grant BF Pang PKT Thomson KS Pickford GE Composition of fluid from the notochordal canal of the coelacanth J Exp Zool 1975 192 165 172 10.1002/jez.1401920206 237059

[B22] Amemiya CT Powers TP Prohaska SJ Grimwood J Schmutz J Dickson M Miyake T Schoenborn MA Myers RM Ruddle FH Stadler PF Complete HOX cluster characterization of the coelacanth provides further evidence for slow evolution of its genome Proc Natl Acad Sci USA 2010 107 3622 3627 10.1073/pnas.0914312107 20139301 PMC2840454

[B23] Noonan JP Grimwood J Danke J Schmutz J Dickson M Amemiya CT Myers RM Coelacanth genome sequence reveals the evolutionary history of vertebrate genes Genome Res 2004 14 2397 2405 10.1101/gr.2972804 15545497 PMC534663

[B24] Kurokawa D Sakurai Y Inoue A Nakayama R Takasaki N Suda Y Miyake T Amemiya CT Aizawa S Evolutionary constraint on Otx2 neuroectoderm enhancers-deep conservation from skate to mouse and unique divergence in teleost Proc Natl Acad Sci USA 2006 103 19350 19355 10.1073/pnas.0604686103 17159156 PMC1748229

[B25] Shashikant C Bolanowski SA Danke J Amemiya CT Hoxc8 early enhancer of the Indonesian coelacanth, *Latimeria *menadoensis J Exp Zool B Mol Dev Evol 2004 302B 557 563 10.1002/jez.b.21018 15470754

[B26] Echelard Y Epstein DJ St-Jacques B Shen L Mohler J McMahon JA McMahon AP Sonic hedgehog, a member of a family of putative signaling molecules, is implicated in the regulation of CNS polarity Cell 1993 75 1417 1430 10.1016/0092-8674(93)90627-3 7916661

[B27] Krauss S Concordet JP Ingham PW A functionally conserved homolog of the Drosophila segment polarity gene hh is expressed in tissues with polarizing activity in zebrafish embryos Cell 1993 75 1431 1444 10.1016/0092-8674(93)90628-4 8269519

[B28] Riddle RD Johnson RL Laufer E Tabin C Sonic hedgehog mediates the polarizing activity of the ZPA Cell 1993 75 1401 1416 10.1016/0092-8674(93)90626-2 8269518

[B29] Osorio J Mazan S Rétaux S Organisation of the lamprey (Lampetra fluviatilis) embryonic brain: Insights from LIM-homeodomain, Pax and hedgehog genes Dev Biol 2005 288 100 112 10.1016/j.ydbio.2005.08.042 16289025

[B30] Tanaka M Münsterberg A Anderson WG Prescott AR Hazon N Tickle C Fin development in a cartilaginous fish and the origin of vertebrate limbs Nature 2002 416 527 531 10.1038/416527a 11932743

[B31] Chang BE Blader P Fischer N Ingham PW Strähle U Axial (HNF3beta) and retinoic acid receptors are regulators of the zebrafish sonic hedgehog promoter EMBO J 1997 16 3955 3964 10.1093/emboj/16.13.3955 9233805 PMC1170019

[B32] Epstein D McMahon A Joyner A Regionalization of Sonic hedgehog transcription along the anteroposterior axis of the mouse central nervous system is regulated by Hnf3-dependent and -independent mechanisms Development 1999 126 281 292 9847242 10.1242/dev.126.2.281

[B33] Ertzer R Müller F Hadzhiev Y Rathnam S Fischer N Rastegar S Strähle U Cooperation of sonic hedgehog enhancers in midline expression Dev Biol 2007 301 578 589 10.1016/j.ydbio.2006.11.004 17157288

[B34] Hadzhiev Y Lang M Ertzer R Meyer A Strähle U Müller F Functional diversification of sonic hedgehog paralog enhancers identified by phylogenomic reconstruction Genome Biol 2007 8 R106 10.1186/gb-2007-8-6-r106 17559649 PMC2394741

[B35] Jeong Y El-Jaick K Roessler E Muenke M Epstein DJ A functional screen for sonic hedgehog regulatory elements across a 1 Mb interval identifies long-range ventral forebrain enhancers Development 2006 133 761 772 10.1242/dev.02239 16407397

[B36] Jeong Y Epstein DJ Distinct regulators of Shh transcription in the floor plate and notochord indicate separate origins for these tissues in the mouse node Development 2003 130 3891 3902 10.1242/dev.00590 12835403

[B37] Jeong Y Leskow FC El-Jaick K Roessler E Muenke M Yocum A Dubourg C Li X Geng X Oliver G Epstein DJ Regulation of a remote Shh forebrain enhancer by the Six3 homeoprotein Nat Genet 2008 40 1348 1353 10.1038/ng.230 18836447 PMC2648611

[B38] Lettice LA Heaney SJH Purdie LA Li L de Beer P Oostra BA Goode D Elgar G Hill RE de Graaff E A long-range Shh enhancer regulates expression in the developing limb and fin and is associated with preaxial polydactyly Hum Mol Genet 2003 12 1725 1735 10.1093/hmg/ddg180 12837695

[B39] Lettice LA Horikoshi T Heaney SJH van Baren MJ van der Linde HC Breedveld GJ Joosse M Akarsu N Oostra BA Endo N Shibata M Suzuki M Takahashi E Shinka T Nakahori Y Ayusawa D Nakabayashi K Scherer SW Heutink P Hill RE Noji S Disruption of a long-range cis-acting regulator for Shh causes preaxial polydactyly Proc Natl Acad Sci USA 2002 99 7548 7553 10.1073/pnas.112212199 12032320 PMC124279

[B40] Müller F Chang B Albert S Fischer N Tora L Strahle U Intronic enhancers control expression of zebrafish sonic hedgehog in floor plate and notochord Development 1999 126 2103 2116 10207136 10.1242/dev.126.10.2103

[B41] Danke J Miyake T Powers T Schein J Shin H Bosdet I Erdmann M Caldwell R Amemiya CT Genome Resource for the Indonesian Coelacanth *Latimeria menadoensis* J Exp Zool 2004 301A 228 234 10.1002/jez.a.20024 14981781

[B42] Goode D Snell P Smith S Cooke J Elgar G Highly conserved regulatory elements around the SHH gene may contribute to the maintenance of conserved synteny across human chromosome 7q36.3 Genomics 2005 86 172 181 10.1016/j.ygeno.2005.04.006 15939571

[B43] Goode DK Snell P Elgar G Comparative analysis of vertebrate Shh genes identifies novel conserved non-coding Mamm Genome 2003 14 192 201 10.1007/s00335-002-3052-z 12647242

[B44] Lemos B Yunes JA Vargas FR Moreira MAM Cardoso AA Seuanez HN Phylogenetic footprinting reveals extensive conservation of Sonic Hedgehog (SHH) regulatory elements Genomics 2004 84 511 523 10.1016/j.ygeno.2004.05.009 15498458

[B45] Müller F Blader P Strähle U Search for enhancers: teleost models in comparative genomic and transgenic analysis of *cis *regulatory elements BioEssays 2002 24 564 572 10.1002/bies.10096 12111739

[B46] Hillier LW Miller W Birney E Warren W Hardison RC Ponting CP Bork P Burt DW Groenen MAM Delany ME International Chicken Genome Sequencing Consortium Sequence and comparative analysis of the chicken genome provide unique perspectives on vertebrate evolution Nature 2004 432 695 716 10.1038/nature03154 15592404

[B47] Murphy WJ Pringle TH Crider TA Springer MS Miller W Using genomic data to unravel the root of the placental mammal phylogeny Genome Res 2007 17 413 421 10.1101/gr.5918807 17322288 PMC1832088

[B48] Steinke D Salzburger W Meyer A Novel relationships among ten fish model species revealed based on a phylogenomic analysis using ESTs J Mol Evol 2006 62 772 784 10.1007/s00239-005-0170-8 16752215

[B49] Bryne JC Valen E Tang MH Marstrand T Winther O da Piedade I Krogh A Lenhard B A S JASPAR, the open access database of transcription factor-binding profiles: new content and tools in the 2008 update Heredity 2008 36 database issue 10.1093/nar/gkm955 PMC2238834 18006571

[B50] Dessaud E McMahon AP Briscoe J Pattern formation in the vertebrate neural tube: a sonic hedgehog morphogen-regulated transcriptional network Development 2008 135 2489 2503 10.1242/dev.009324 18621990

[B51] Tajima F Simple methods for testing the molecular evolutionary clock hypothesis Genetics 1993 135 599 607 8244016 10.1093/genetics/135.2.599 PMC1205659

[B52] Muller F Albert S Blader P Fischer N Hallonet M Strahle U Direct action of the nodal-related signal cyclops in induction of sonic hedgehog in the ventral midline of the CNS Development 2000 127 3889 3897 10952887 10.1242/dev.127.18.3889

[B53] Chiu CH Dewar K Wagner GP Takahashi K Ruddle F Ledje C Bartsch P Scemama JL Stellwag E Fried C Prohaska SJ Stadler PF Amemiya CT Bichir HoxA cluster sequence reveals surprising trends in ray-finned fish genomic evolution Genome Res 2004 14 11 17 10.1101/gr.1712904 14707166 PMC314268

[B54] Taylor JS Braasch I Frickey T Meyer A Van de Peer Y Genome duplication, a trait shared by 22,000 species of ray-finned fish Genome Res 2003 13 382 390 10.1101/gr.640303 12618368 PMC430266

[B55] Ghanem Nl Jarinova O Amores A Long Q Hatch G Park BK Rubenstein JLR Ekker M Regulatory roles of conserved intergenic domains in vertebrate Dlx bigene clusters Genome Res 2003 13 533 543 10.1101/gr.716103 12670995 PMC430168

[B56] Du SJ Dienhart M Zebrafish *tiggy-winkle hedgehog *promoter directs notochord and floor plate green fluorescence protein expression in transgenic zebrafish embryos Dev Dyn 2001 222 655 666 10.1002/dvdy.1219 11748834

[B57] Dahn RD Davis MC Pappano WN Shubin NH Sonic hedgehog function in chondrichthyan fins and the evolution of appendage patterning 2007 445 311 314 17187056 10.1038/nature05436

[B58] Sagai T Amano T Tamura M Mizushina Y Sumiyama K Shiroishi T A cluster of three long-range enhancers directs regional Shh expression in the epithelial linings Development 2009 136 1665 1674 10.1242/dev.032714 19369396

[B59] Sagai T Hosoya M Mizushina Y Tamura M Shiroishi T Elimination of a long-range cis-regulatory module causes complete loss of limb-specific Shh expression and truncation of the mouse limb Development 2005 132 797 803 10.1242/dev.01613 15677727

[B60] Sagai T Masuya H Tamura M Shimizu K Yada Y Wakana S Gondo Y Noda T Shiroishi T Phylogenetic conservation of a limb-specific, *cis*-acting regulator of Sonic hedgehog (Shh) Mamm Genome 2004 15 23 34 10.1007/s00335-033-2317-5 14727139

[B61] Feinberg AP Vogelstein B A technique for radiolabeling DNA restriction endonuclease fragments to high specific activity Anal Biochem 1983 132 6 13 10.1016/0003-2697(83)90418-9 6312838

[B62] PHRED http://www.phrap.org

[B63] UCSC genome browser http://genome.ucsc.edu

[B64] Stamatakis A Hoover P Rougemont J A rapid bootstrap algorithm for the RAxML Web servers Syst Biol 2008 57 758 771 10.1080/10635150802429642 18853362

[B65] Repeatmasker http://www.repeatmasker.org

[B66] Frazer KA Pachter L Poliakov A Rubin EM Dubchak I VISTA: computational tools for comparative genomics Nucl Acids Res 2004 32 W273 279 10.1093/nar/gkh458 15215394 PMC441596

[B67] Brudno M Do CB Cooper GM Kim MF Davydov E Program NCS Green ED Sidow A Batzoglou S LAGAN and Multi-LAGAN: efficient tools for large-scale multiple alignment of genomic DNA Genome Res 2003 13 721 731 10.1101/gr.926603 12654723 PMC430158

[B68] Subramanian A Kaufmann M Morgenstern B DIALIGN-TX: greedy and progressive approaches for segment-based multiple sequence alignment Algorithms Mol Biol 2008 3 6 10.1186/1748-7188-3-6 18505568 PMC2430965

[B69] Tamura K Dudley J Nei M Kumar S MEGA4: Molecular Evolutionary Genetics Analysis (MEGA) software version 4.0 Mol Biol Evol 2007 24 1596 1599 10.1093/molbev/msm092 17488738

[B70] Hamburger V Hamilton HL A series of normal stages in the development of the chick embryo J Morphol 1951 88 49 92 10.1002/jmor.1050880104 24539719

