# Supplementary material for: Conservation of shh cis-regulatory architecture of the coelacanth is consistent with its ancestral phylogenetic position
Source: EvoDevo. 2010 Nov 3;1:11. doi: 10.1186/2041-9139-1-11 (PMC2992049; doi:10.1186/2041-9139-1-11)
Supplement: Additional file 1 — DNA sequence alignments. Alignments of shh enhancers ar-A, ar-B, ar-C, and ar-D with putative transcription factor binding sites. [file 2041-9139-1-11-S1.PDF]

ar-D

```
                   IRX                                                       GATA2 / Homeobox
          ....|....|....|....|....|....|....|....|....|....|....|....|....|....|....|....|....|....|
Human     AAG-AAAACATGTACACAACATTCAAACACCGTCTGCCACTG-GAAGAGACT-------CCAGGACAATTGTGCACATACTTGGCCTGCT
Mouse     AAGAAAAACATGTACACAACATTCAAACACTGTCTGCCAATGTGAAGAAACAAGAGAA-TCGAGACAATCACACGCATACTTGGCCTGCT
Opossum   AAAGAAAACATGTATACAACATTCAAAAATTGGCTGCCAATGGAAAGAAACAAGAGAAACAAAGACAATCCTGCACATACTTTGCCTGCT
Chick     AAAGAAAACATGTAAACTGTGCTCAAAAATAGTCTGCAAATGTAAGGAAACAAGAAAAACTAAGACAATCATGCACATACCTCTCCTGCT
Latimeria ---AAAAACATGTGTACTGTGTTTAAAAGTAGTCTGCAAATGTAAGAAAACAAGAGAAATTGGGACAATTATCCACATACCTCGCTTGCT
Zebrafish ---------------------------------------------GAAAACAAGAGAAAATAAGACAATGAAACACATACCTTACCCGCT

                                                            Pax2 Nkx3-2
          ....|....|....|....|....|....|....|....|....|....|....|....|....|....|....|....|....|....|
Human     ACCATGGCCCGGCGAATTCAGCGGTTACCATGGCGGGGTAGAGCCTGGAGGAGGACTCCAGAGGGCAGGCTTTGT--GTGGGCACTGCTC
Mouse     CCCATGGTCCAGTGAATGCCAGAGTTACCATGGTGGGAGGGAGC--AGAGGAGCATCCCCGAGGGCAGGCTTTGT--CTGGGTGCTACTC
Opossum   ACCATGGACCACTGAATTCAACGGTTACCATGGTGGGCCCTAGCTGGAAGGTGGACTCTAGAGGGCAAGCTTTGT--TTCTGCTCTGCCC
Chick     GCCATGGCCCAGTGAATACAGTGGTTACCATGGCTGGATCTGGCTACGAGGTGGACTTTAGAGGGCAAGCTTTGTTCCTGATCTCTGCCC
Latimeria ACCATGGACCAGTGAATACAGTGGTTACCATGGCTAGATCTGGCTATGAGGTGGACTTTGGAGGCCAAGCTTTGTTTCTGATCTCTCCCC
Zebrafish GCTATGGGCCAGGGAACACAGTGGTTACCATGGCTTAATCTGTCACTGAAGTGGACTTTA------------------------------

                                         FoxA2                                  ETS1
          ....|....|....|....|....|....|....|....|....|....|....|....|....|....|....|....|....|....|
Human     AAACAGAGAGCTGCGATTCAGTCTGTTGGCTTGTGTACTTTGGTGTGAGCCATCCTCACGCT------GAAAAGGCCAAGAGGAGAAGTG
Mouse     AAACAGAAAGCTG-GATTCA-TCTGAGGGTCTGTGTACTTTGGGGCAAGCCA-CCTCACACT------GAAAAGGCCA-GGGGAGAAGTG
Opossum   AAACAGAGAGCCCTGATTCAGTCTGTTGGTTTGTGTACTTTGGGGTAAGCCATGCTCACACT------GAAAAAGCCAAGGGGAGATCAT
Chick     AAACAGAAAGCCTTGATCCAGTCTGTTGGTTTGTGTACTTTGGGC-AAGCCAACCTTACACT------GCAAAAGGCA-------GCAAG
Latimeria AAACACAAAGCCCTGATCCAAGCTACAGGTTTGTGTACTTTGGAG-AAGCCAACTCCAGATCACAGCAGCAAAAGGAAAAAAAAA----C
Zebrafish ------------------------------------------------------------------------------------------

                   FOXL1
          ....|....|....|....|....|....|....|....|....|....|
Human     TTGA---GTTTGACTTGATCTCATGCCTTTAGGTTTCTCTTGACATCCCG
Mouse     --GA---GTTTGGCTCCTTCTCATACCTTTGGGTCTCTCTTGGCATCCTG
Opossum   TTTA---GCTTGACTTTATCTCATACCTTTGAGTTTCTCTTAGCATCCTA
Chick     TTAA---GTTTGAATTTATTTCATGCTTTCAGGTTTCTCTTAGCATCCCC
Latimeria TTTA---GTTTGAGTTTATATAGTGTCTT-AGGTTTCTGTTGACACCTCA
Zebrafish -------------------------------------------
```

ar-A

```
                                                               FOX A1/A2           Nkx2-5 ETS1/SPIB
          ....|....|....|....|....|....|....|....|....|....|....|....|....|....|....|....|....|....|
Latimeria ------GAAATCATTGAGCCTGGCATTTAAGAATCCCACCAGGTTGTTTACACTGACAGGTACCTGTTAAGTGTTTCTTTTAAGCAATT-
Chick     ---GAACTCAGAGATCAGCTTGGCATTTAGGAAGCCCACCAGGTTGTTTACACTGACAGGTACCTGTTAAGTGTTTCCTTCAAGCAATT-
Opossum   -----------------------------GGAAGCCCACCAAGTTGTTTACAGTGACAGGTACCTGCTAAGTGTTTCCTCCGAGCATTCA
Human     ------------------------------------------------------------------------------------------
Mouse     ------------------------------------------------------------------------------------------
Zebrafish GAAGCTCGCGAAATTAGGCGCAACTGCGAGGGTATCCAGGAGGCTGTTTGCTCTGACAGGTAGCTGATAAGTGTTTCCTCTAAAACAGTT
Medaka    --------------------------------GAGATGGGAGTTTGTTTGCACTGACAGGTAGCTGACAAGTGTTTCCTTTAACCCAAT-
Fugu      --------------------------------GAGCTGGCGGCCTGTTTGCACTGACAGGTAGCTGATAAGTGTTTCCTCTAACTAAGT-

          ....|....|....|....|....|....|....|....|....|....|....|....|....|....|....|....|....|....|
Latimeria --CATTTGTGAAGAGATTAGGAGCT-GGCAGTGAG--GGC---TTGTTGAACAA-CTTTGTCTC----AGCATAAAGCGTTAGCTGAAGA
Chick     --CATTTGTGAAGAGATTAGGAGCTGGCAGGAGAG--GGC---TTGTTGAACAA-CTTCGCCTCC----ACACAAAGCGTCGGCGGAGGC
Opossum   TTTGTGTAGAGAGAGATTAGGCGCTTGGCAAAGAGCTGGGG-CGTGTTGAACAA-CTTTGCCTCAGCTGACACAAAAGCCCGAGCTGAGG
Human     --GATCAGGGAGGTAGGCGGGAGCTCAGGCG-------------TGGGGAACAA-CTTGGCCTCCGCCGACACAAAGC-CCGGCCCCGGC
Mouse     --GATCCGGGAGGTGA------GCT-GGAGGC-----------TTCAGGAACAA-CTTGGCCTCCGCAGACACAAAGC-TGGGCCCCAGT
Zebrafish C-ATTTTGGGGGGAGATTAGTTGCT-GACTGTGACTAGGCGGCTTGTTGAACAAACCTCGTGTGG---AGCACAAAGCGGCGGCACAGGG
Medaka    --CATTTGTGAGAAGATTA---AGT-GGGCAGGC----------TGTTGAACAA-CTTTACGAGG---AGCCCAG-----CGGTCCAAAG
Fugu      --AATTTGTGAAAAGATTA---AGA-GAG-AGGC----------TGTTGAACAA-CTTTACCCCG---AGCACAAAGTGCTGGTCTGGGG

                                                                            Homeobox
          ....|....|....|....|....|....|....|....|....|....|....|....|....|....|....|....|....|....|
Latimeria AAC-CCTGCTGGG-TTCACGGTGGCTGAACAGAATTGGGGTTGATTTGCGGCACACGACCCAATGAATTAATAAATAGTTTAGGCTTTTC
Chick     AGC-CCTGCTGAGCTTCACGGTGGCTGAACAGAATTGGGCTTGATTTGCGGCACACGACCCGATGAATTAATAAATAGTCTGGGCTTCAC
Opossum   CGGCCCTGCTGGGCTTCACGGTGAGTGAACAGAATTGGGCTTGATTCGTGGCACACGACCCAGTGAATTAATAAATTGTCTGAGCTTCAC
Human     GGC-CCTGCTGGGCTTCACGGTGGCTGCACAGAGTCGGGCTTGATTCGCGGCACACGACCCAATGAATTAATAACCGGCCTGGGCTTCC-
Mouse     GAC-CCTGCTGGGCTTCACGGTGGCTGCACAGAGTCGGGCTTGATTAGCGGCACACGACCCAATGAATTAATAACCGGGCTGAGCTTCAA
Zebrafish GGTTCCCGCTGCGCTTCATGGTGGCTGAACAGAATTTGGCTTGATTTGCGGCACACGACTCAATGAATTAATAAAGAGTTCAGGCTTCAC
Medaka    CGC-CCCGCTGGGCTTCATGGTGGCTGAGTAGAATTTGGCTTGATTCGTGGCACACGACCTGATGAATTAATAAAAGGATAAAGCTTTGC
Fugu      CGC-CCCGCTGGGCTTCACGGTGACTGAGCAGAATTTGGCTTGATTCGCGGCACACGACCTAATGAATTAATAAATAGGTTAAGCTTTAC

          ....|....|....|....|....|....|....|....|....|
Latimeria ACTCAGACAATTCAGAT-TCCCCCTGTACTAG--------------
Chick     GGCTTTTGACTCCGACAATCCAGCTCAGGCGGAGTGAAAGGGAGG
Opossum   GGCTTTTGACTCCGACAATCCAGCTCAGCCAGTATAGAAGAAAG-
Human     CGGCTTTGCCTGCGACAATCCCGCCCAGCGCGGGCGGAGGAGAGG
Mouse     TTGCCTTGCCTGCGACATTCCCTCCCATCGCTGGCGGGGAGTAC-
Zebrafish GGCTCATCAA--------TCCCCGGTAG-----------------
Medaka    ACCGCTT--------------------------------------
Fugu      GGCTCTTGACTCTGAAAATCCCTCTCAGAATGCGTTTT-------
```

ar-B

```
                       Sox5         FOXL1                             BRCA1  HIF1A::ARNT
                  ....|....|....|....|....|....|....|....|....|....|....|....|....|....|....|....|....|....|
zebrafish         CATATT-TTGAATAGATTTTATATAACCTCTGAAAGACCGTCAGT--T-AAGCAATCACATCACGTCCC-TTGAGCAAACCTGTGCTGCA
Fugu arB 5        ------------------------------GGGAGCAGCCCAACT--T-AAGGAGGCACGTCACGTCCC----AGAAGACTT-TGCGGGC
Medaka arB        ------------------------------AAAAACACCTCAACT--T-AAGCAAGCAAGTCACGTTTC----AG--GACTT-TGCCGG-
Latimeria         TGTATTGTTGTTTTTATGGTTGAGAAACGCACATTTCAGCTTGCCTGTTAAGCAACCCTGTCGCGTCCT-----TACAAGTAGTGCTTCA
opossum ar        TATATTACCGTTTTTATTGCTGCCAAATACACAGAAC--TTTTCCTGTTAAGCAATCTTGTCAAGTCCCCTTGAAAACTGTGGAGTAAGA
opossum ar        ------------------------------------------------------------------------------------------
Fugu arB 3        ------------------------------------------------------------------------------------------
Medaka arB        ------------------------------------------------------------------------------------------

                                                                                             FOXL1     Nkx2-5
                  ....|....|....|....|....|....|....|....|....|....|....|....|....|....|....|....|....|....|
zebrafish         A-GA---ATAATGAGTAGTTGCTTCTCCTCT--------TGGACGCAGTGAAAGAGTCTTTTGTGGTGTTTTATTTGACATATTGGTTAA
Fugu arB 5       A-GACGCATAATGAA-AGTTTC-CCTCCG----------TCGTTGCACTGAAAAGCCTCTTTGTGATGTTTTATTTGACATATTGGTCGA
Medaka arB        A-GAGG-ATAATGAA-CGTTTTTCCTTCGCTTTTTTTTCTTTTTGCACTGAAA-GCCTCTTTGTGATGCTTTTTTTTCCAAATTGGTTGG
Latimeria         A-GA---ATAATGCA-AGTTTCTTTTCCCTT-------CTGGGAGCTGTGAAAAGGGCCTTTGTGATGTTTTATTTTACATATTGGTAAA
opossum ar        ATAA---TATAAGGATTCTCCGACTCCTTCCAAC--------------------------------------------------------
opossum ar        -----------------------------------CAAGCTGTTGAA--AAAAGGATCCTTTGTGGTTGAGGATTTTACATATTGGTAAC
Fugu arB 3        ------------------------------------------------------------------------------------------
Medaka arB        ------------------------------------------------------------------------------------------

                    BRCA1
                  ....|....|....|....|....|....|....|....|....|....|....|....|....|....|....|....|....|....|
zebrafish         GTG-TTGGGAAGAGTTGTCCTGGTGAAGGCCAAGCATCTCAAAGC-AAGGTCCCGGGAAAGGTTAGCACCCAAACATGGCCTGATATTCC
Fugu arB 5       GCG-TCCAGAATAGTTGTCTTGGAGAAGGCCAAGCATCC---------------------------------------------------
Medaka arB        GCG-TCAAAAATAGTTGTCTTGTAGAAGGCCAAACATCT---------------------------------------------------
Latimeria         GTC-TTGGGAAGAGTTGTCCTGGAGAAGGCCAAGCATCTCAACTC-AGAGTCGCAGCAAAGGTTAGAGAGTGCATT--------------
opossum ar        ------------------------------------------------------------------------------------------
opossum ar        CTCTTTGGGGAGAGTTGTCTGGGAGAAGGCCAAGCATC-CT-CTCTAGAATT--GGGAAAAGTTAAAGCAAACCAT--------------
Fugu arB 3        ------------------------------------------------------------------------------------------
Medaka arB        ------------------------------------------------------------------------------------------

                  ....|....|....|....|....|....|....|....|....|....|....|....|....|....|....|....|....|....|
zebrafish         ACAGTTCATACCTGCACCTCAGTGAGACCTACTGCTCTAAATCTCAAGGTTATAATAATGAACACCACTAACCGAATTACTTTTTTTACC
Fugu arB 5       ------------------------------------------------------------------------------------------
Medaka arB        ------------------------------------------------------------------------------------------
Latimeria         ------------------------------------------------------------------------------------------
opossum ar        ------------------------------------------------------------------------------------------
opossum ar        ------------------------------------------------------------------------------------------
Fugu arB 3        ------------------------------------------------------------------------------------------
Medaka arB        ------------------------------------------------------------------------------------------

                                                                                 ETS1        GATA2
                  ....|....|....|....|....|....|....|....|....|....|....|....|....|....|....|....|....|....|
zebrafish         CAATTTCATTGTATGCATTCTGGCCTTTTAAAGCAAAGACATGGCCCCTGATTCAGTCACACCTTTCCCTGGTTGGA---TCTATACGTT
Fugu arB 5       ------------------------------------------------------------------------------------------
Medaka arB        ------------------------------------------------------------------------------------------
Latimeria         ------------------------------------------------------------------------------------------
opossum ar        ------------------------------------------------------------------------------------------
opossum ar        ------------------------------------------------------------------------------------------
Fugu arB 3        --------------------------------------------------------CCCTACCTTTCCCACACCAGAGAACCTTCATGTT
Medaka arB        --------------------------------------------------------CGCTGCTTTTCCCGAGACTGAAGCCCCACATGTT

                                           Nkx2-5                            GATA3/2/ETS1
                  ....|....|....|....|....|....|....|....|....|....|....|....|....|....|....|.
zebrafish         TTAGATCAGTGAAGCGAATGCAGTAACTTATAAATTTTTCATTG--AGCTCCTTGAGC--AGAT-TATCCTTGCAT
Fugu arB 5       ----------------------------------------------------------------------------
Medaka arB        ----------------------------------------------------------------------------
Latimeria         ----------------------------------------------------------------------------
opossum ar        ----------------------------------------------------------------------------
opossum ar        ----------------------------------------------------------------------------
Fugu arB 3        ATAGACCAGTGAAGCGAACTCGGTGCCTCATTAATATTTCATCAGCAGCTTTCTTATCCGAGATGTGCTCTTT---
Medaka arB        ATATGCCAGTGAAGCGAATGCGGTGCTTCATCCATATTTCATGAGCAGCTTTCTTACCCGAGATGTGCCCTCT---
```

ar-C

```
           C1
           ....|....|....|....|....|....|....|....|....|....|....|....|....|....|....|....|....|....|
Human      -TTGCACCTGAGCAAATAGGGAGGGGGAGGCCCGCGAGCTGGGGAGAGAGTGAGCTG-----AGAACA--GGGAGGGGAGAAAATGGAAG
Mouse      -TTGCACCTGAGCAAATAGGGAGGGGGCGGCCAGCGAGCTGt----AGAGTGAGCTG-----AGAATG--GGGAGAGGgggt--TGGAAG
Opossum    TCTGCACCCGAACCAAatatCGGGG--------------TGGGTAGTGAGAGAGtgccaaaaAAAAGA--AGAGGGGGAGAAAATGGAAG
chick      TCTGCACCTGAACAAATAGGGAGGGGGAGaaaag-------------------GGGG-----GGAAGA--AGCTGGGAAAAAAATGGAAG
Latimeria  TCTGCACCTGAGCAAATAGGGA------------------------------------------AAGA--AGCTGGGAAAA--GTGGAAG
Zebrafish  GCTGCACCTGAGCAAATATGAA------------------------------------------AgaggcgcaaGGGAAAG--GCAGAAG
Fugu       TCTGCACCTGAGTAAATATGGG------------------------------------------AAGAGTCGCTGGGAAAG--GCGTAAG
Medaka     TCTGCACCTGAGTAAATATGGG------------------------------------------GAGAGTCGCTGGGAAAG--GCGTAAG
```

```
           C2                                      C3                             C4
                              GATA2    GATA2
           ....|....|....|....|....|....|....|....|....|....|....|....|....|....|....|....|....|....|
Human      TGT--CCCCTTCCAAGAGTGTCTCCTGTTTATCCCA-GAAATCACAATGACAATGC-TG----------GGCCCTTTATTGGATTTT---
Mouse      TATCCCCTCTTCCGAGGCTGTCTCCTATTTATCCCA-CAAATCACAATGACAATAT-CCcacaataccaAGCTCTTTATTGGATTTT---
Opossum    CGCCCTCTCTTCTAAGAGTGCCTCCCATTTATTCAAaGGAATCGAAATGACAATGCgTG----------AGTTCTTTATTAGATTTT---
chick      TGTCCTCTCTTCCAAGAGTGTCTGC-ATTTATTACA-TGAATCAGAATGACAATGC-TG-----------ACCCTTTATTGGATTTT---
Latimeria  TACCCTCTCTTCCAAAAGTATCTTC-ATCCATTAGA-TAAATCAGAATGACAATGC-TG-----------ATTCTTTATTGGATTTT---
Zebrafish  TGT--CCTTTTCCAAGAGT-GCTCT-GTACACAAGC-TGCATTAGAATGACAATGT-CC-----------GGCCTTTATTGGTTTTT---
Fugu       TGT--TCTTTACCGAGAGCAGCTCC-ATCCACAGGC-TGCTTTAGAATGACAATGG-CC-----------GCCCTTTATTGGGTTTTTT-
Medaka     TGT--TCTTTGCCGAGAGTCGCTGG-ATCCACAGGA-TGATTTAGAATAACAATGC-CT-----------TCCCTTTATTGAGTTTTTTt
```

```
          Homeobox   Foxh1                           T-box                    Foxa2
           ....|....|....|....|....|....|....|....|....|....|....|....|....|....|....|....|....|....|
Human      -AATTAGAAAATCCACACAAGCCTCG------------GATTTTCACACCTCGGCCAAT--CTCTGGAATGTTTGTCCAGTTGCTACAA-
Mouse      -AATTAGAAAATCCACACAAGTCTCG------------GGCTTTCACACCTCGGGCAAA-CCTCTGGAATGTTTGTCCAGTTGCTACAA-
Opossum    -AATTAGAGAACCCAAACAAATGCCG------------GGTTTTCACTCCCCAGACCAAtTCCTTGGTGTGTTTGGAGAGTTGCTACAAa
chick      -AATTAGAGAACCCAAACAAGCGCTGCTCAAAGAAGCAGATTTTCACACCTCAGCCAAT-TCACTGGCATGTTTGTAGAGCTGCTACA--
Latimeria  -AATTAGGGAGCCtgAGCTACCAATTCTCCACACAGAAGATTTTCACACCTCTGTCTAT-TCACTGACATGCTTAAAG-GGTTCTGAA--
Zebrafish  -AATTAGA----------------------------------------------------------------------------------
Fugu       -AATTA------------------------------------------------------------------------------------
Medaka     aAATTA------------------------------------------------------------------------------------
```

```
           ....|....|....
Human      CTACTGCAGCTATT
Mouse      CGACTACTGCTATA
Opossum    CTATTCCAGTTATT
chick      CTACTAGATCTATT
Latimeria  CAACTAGCTTTATT
Zebrafish  --------------
Fugu       --------------
Medaka     --------------
```

Footnote:

Elements C1,C2, C3 and C4 indicated in the ar-C alignment were taken from Hadzhiev et al. (2007) (34), putative transcription factor binding sites in grey were taken from Jeong et al. 2003 (36).
