# Supplementary material for: Conservation of shh cis-regulatory architecture of the coelacanth is consistent with its ancestral phylogenetic position
Source: EvoDevo. 2010 Nov 3;1:11. doi: 10.1186/2041-9139-1-11 (PMC2992049; doi:10.1186/2041-9139-1-11)
Supplement: Additional file 2 — ar-B enhancer conservation. VISTA plot of the ar-B specific genomic region. Shuffle-LAGAN alignment, visualized with mVISTA. [file 2041-9139-1-11-S2.PDF]

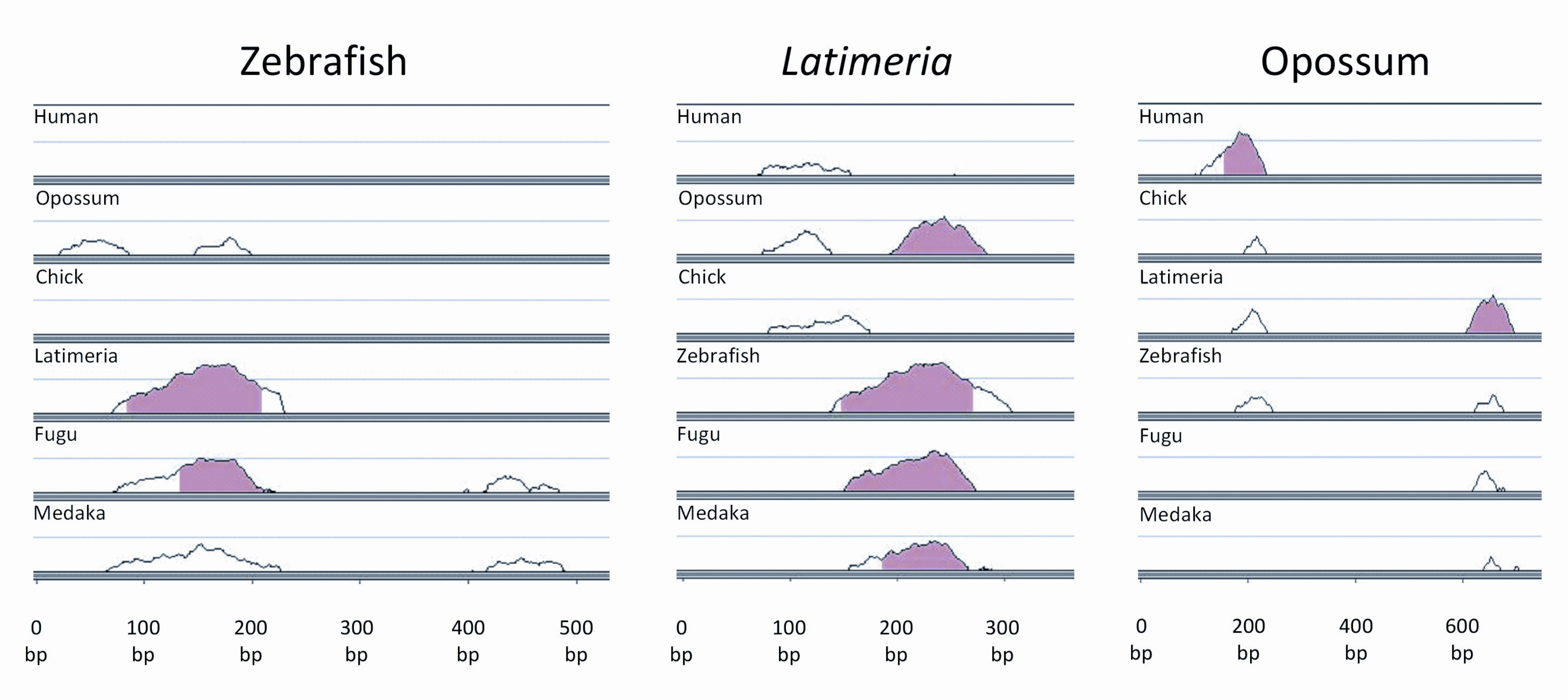

***ar-B* enhancer conservation**. Shuffle-LAGAN alignment, visualized with mVISTA. In opossum, the sequence that retains *ar-B* conservation is interrupted with approximately 230 bp of repetitive DNA. The 5´- part of this region is further conserved in *shh* loci of placental mammalian species, such as human.
