# Supplementary material for: Conservation of shh cis-regulatory architecture of the coelacanth is consistent with its ancestral phylogenetic position
Source: EvoDevo. 2010 Nov 3;1:11. doi: 10.1186/2041-9139-1-11 (PMC2992049; doi:10.1186/2041-9139-1-11)
Supplement: Additional file 3 — VISTA plot of the shh genomic region. VISTA plot with zebrafish shh as reference sequence. Shuffle-LAGAN alignment, visualized with mVISTA. This figure is used for indication of the putative ar-E CNE that is found in zebrafish, medaka and fugu. [file 2041-9139-1-11-S3.PDF]

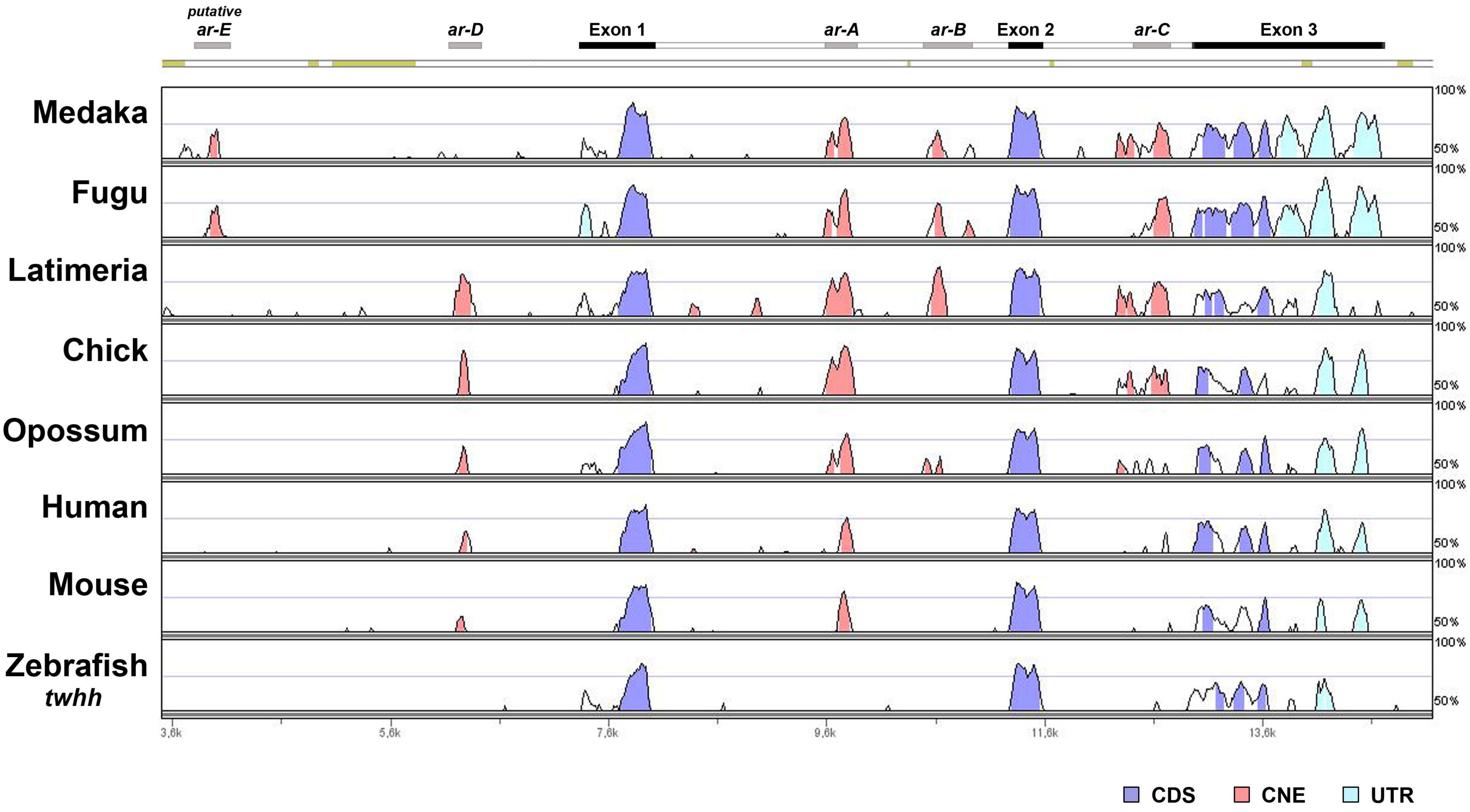

VISTA plot of the *shh* genomic region (*Danio rerio shh* as reference sequence). Shuffle-LAGAN alignment (repeatmasker applied), visualized with mVISTA. The crossbar indicates the deduced *shh* gene structure (black: exons). Enhancer locations are indicated by grey bars as well as the putative *ar-E* CNE. Masked repeats are indicated by yellow bars. Conservation parameters were set as: Min Y: 50, min Id: 70, Min length: 60.
