# Supplementary material for: Conservation of shh cis-regulatory architecture of the coelacanth is consistent with its ancestral phylogenetic position
Source: EvoDevo. 2010 Nov 3;1:11. doi: 10.1186/2041-9139-1-11 (PMC2992049; doi:10.1186/2041-9139-1-11)
Supplement: Additional file 4 — enhancer blocks. Conserved enhancer sequences used for Relative Rate Tests are presented. [file 2041-9139-1-11-S4.PDF]

ar-D / SFPE1

```
                     10        20        30        40        50        60        70        80        90       100
           ....|....|....|....|....|....|....|....|....|....|....|....|....|....|....|....|....|....|....|....|
Human      -GTCT-GCCACTGGA--------AGAGACTCCAGGACAATTGTGCACATACTTGGCCTGCTACCATGGCCCGGCGAATTCAGCGGTTACCATGGCGGGGT
Opossum    TGGCT-GCCAATGGAAAGAAACAAGAGAAACAAAGACAATCCTGCACATACTTTGCCTGCTACCATGGACCACTGAATTCAACGGTTACCATGGTGGGcc
Mouse      TGTCT-GCCAATGTGAAGAAACAAGA-NAATCGAGACAATCACACGCATACTTGGCCTGCTCCCATGGTCCAGTGAATGCCANAATTACCATGGTGGGG-
Chick      AGTCT-GCAAATGTAAGGAAACAAGAAAAACTAAGACAATCATGCACATACCTCTCCTGCTGCCATGGCCCAGTGAATACAGTGGTTACCATGGCTGGAT
Latimeria  AGTCT-GCAAATGTAAGAAAACAAGAGAAATTGGGACAATTATCCACATACCTCGCTTGCTACCATGGACCAGTGAATACAGTGGTTACCATGGCTAGAT
Zebrafish  AGTCTaGAAAATGTGTGAAAACAAGAGAAAATAAGACAATGAAACACATACCTTACCCGCTGCTATGGGCCAGGGAACACAGTGGTTACCATGGCTTAAT

                    110       120       130       140       150       160       170       180       190       200
           ....|....|....|....|....|....|....|....|....|....|....|....|....|....|....|....|....|....|....|....|
Human      AGAGCCTGGAGGAGGACTCCAGAGGGCAGGC-----TTTGTGT-GGG--CACTG-CTCAAACAGAGAGC--TGCGATTCAGTCTGTTGGCTTGTGTACTT
Opossum    CTAGCTGGAAGGTGGACTCTAGAGGGCAAGC-----TTTGTTT-CTG--CTCTG-CCCAAACAGAGAGCCCTG--ATTCAGTCTGTTGGTTTGTGTACTT
Mouse      --AGGGAACANAAGAACTCCCCGAGGCAGGC-----TTTGTCT-GGG--TGCTA-CTCAAACAGAAAGC--TG-gaTTCA-TCTGAGGGTCTGTGTACTT
Chick      CTGGCTACGAGGTGGACTTTAGAGGGCAAGC-----TTTGTTC-CTGATCTCTG-CCCAAACAGAAAGCCTTG--ATCCAGTCTGTTGGTTTGTGTACTT
Latimeria  CTGGCTATGAGGTGGACTTTGGAGGCCAAGC-----TTTGTTT-CTGATCTCTC-CCCAAACACAAAGCCCTG--ATCCAAGCTACAGGTTTGTGTACTT
Zebrafish  CTGTCACTGAAGTGGACTTTAAGGGGtgacagggtttTTGCAGGCTGATCTTTCtCCCAAACAGACAGCTT--------------AGCATTTGTGTGCAC

           ...
Human      TGG
Opossum    TGG
Mouse      TGG
Chick      TGG
Latimeria  TGG
Zebrafish  AGG
```

ar-A

```
                     10        20        30        40        50        60        70        80        90       100
           ....|....|....|....|....|....|....|....|....|....|....|....|....|....|....|....|....|....|....|....|
Human      c-AGGCGTGGGGAACAACTTGGCCTC---CGCCGACACAAA-G-CCCGGCCCCGG-CGGCCCTGCTGGGCTTCACGGTGGCTGCACAGAGTCGGGCTTGA
Mouse      GGAGGCTTCAGGAACAACTTGGCCTC---CGCAGACACAAA-G-CTGGGCCCCAG-TGACCCTGCTGGGCTTCACGGTGGCTGCACAGAGTCGGGCTTGA
Opossum    tGGGGCGTGTTGAACAACTTTGCCTC---AGCTGACACAAAAGCCCGAGCTGAGG-CGGCCCTGCTGGGCTTCACGGTGAGTGAACAGAATTGGGCTTGA
Chick      GAGGGCTTGTTGAACAACTTCGCCTC------CACAC-AAA-GCGTCGGCGGAGG-CAGCCCTGCTGAGCTTCACGGTGGCTGAACAGAATTGGGCTTGA
Latimeria  GAGGGCTTGTTGAACAACTTTGTCTC------AGCAT-AAA-GCGTTAGCTGAAG-AAACCCTGCTGGG-TTCACGGTGGCTGAACAGAATTGGGGTTGA
Zebrafish  GGCGGCTTGTTGAACAAa----CCTCgtGTGGAGCAC-AAA-GCGGCGGCACAGGgGGTTCCCGCTGCGCTTCATGGTGGCTGAACAGAATTTGGCTTGA
Medaka     GCAGGCT-GTTGAACAACTTTAC-----GAGGAGCCC-A---GCGGTCC---AAA-GCGCCCCGCTGGGCTTCATGGTGGCTGAGTAGAATTTGGCTTGA
Fugu       AGAGGCT-GTTGAACAACTTTACCCC--GAGCAC----AAA-GTGCTGGTCTGGG-GCGCCCCGCTGGGCTTCACGGTGACTGAGCAGAATTTGGCTTGA

                    110       120       130       140       150
           ....|....|....|....|....|....|....|....|....|....|.
Human      TTCGCGGCACACGACCCAATGAATTAATAACCGGCCTGGGCTTCCCGGCTT
Mouse      TTAGCGGCACACGACCCAATGAATTAATAACCGGGCTGAGCTTCAattgcc
Opossum    TTCGTGGCACACGACCCAGTGAATTAATAAATTGTCTGAGCTTCACGGCTT
Chick      TTTGCGGCACACGACCCGATGAATTAATAAATAGTCTGGGCTTCACGGCTT
Latimeria  TTTGCGGCACACGACCCAATGAATTAATAAATAGTTTAGGCTTTTCACtca
Zebrafish  TTTGCGGCACACGACTCAATGAATTAATAAAGAGTTCAGGCTTCACGGCTC
Medaka     TTCGTGGCACACGACCTGATGAATTAATAAAAGGATAAAGCTTTGCACCGC
Fugu       TTCGCGGCACACGACCTAATGAATTAATAAATAGGTTAAGCTTTACGGCTC
```

ar-C

```
                     10        20        30        40        50        60        70        80        90       100
           ....|....|....|....|....|....|....|....|....|....|....|....|....|....|....|....|....|....|....|....|
Human      GGAGGGGAGAAAATGGAAGTGTCCC--CTTCCAAGAGTGTCTCCTGTTTATCCCA-GAAATCACAATGACAATGC-TG----------GGCCCTTTATTG
Mouse      GGAGAGGgggt--TGGAAGTATCCCCTCTTCCGAGGCTGTCTCCTATTTATCCCA-CAAATCACAATGACAATAT-CCcacaataccaAGCTCTTTATTG
Opossum    GAGGGGGAGAAAATGGAAGCGCCCTCTCTTCTAAGAGTGCCTCCCATTTATTCAAaGGAATCGAAATGACAATGCgTG----------AGTTCTTTATTA
chick      GCTGGGAAAAAAATGGAAGTGTCCTCTCTTCCAAGAGTGTCTGC-ATTTATTACA-TGAATCAGAATGACAATGC-TG-----------ACCCTTTATTG
Latimeria  GCTGGGAAAA--GTGGAAGTACCCTCTCTTCCAAAAGTATCTTC-ATCCATTAGA-TAAATCAGAATGACAATGC-TG-----------ATTCTTTATTG
Zebrafish  caaGGGAAAG--GCAGAAGTGT--CCTTTTCCAAGAGT-GCTCT-GTACACAAGC-TGCATTAGAATGACAATGT-CC-----------GGCCTTTATTG
Fugu       GCTGGGAAAG--GCGTAAGTGT--TCTTTACCGAGAGCAGCTCC-ATCCACAGGC-TGCTTTAGAATGACAATGG-CC-----------GCCCTTTATTG
Medaka     GCTGGGAAAG--GCGTAAGTGT--TCTTTGCCGAGAGTCGCTGG-ATCCACAGGA-TGATTTAGAATAACAATGC-CT-----------TCCCTTTATTG

                    110       120
           ....|....|....|....|
Human      GATTTT----AATTAGAAAA
Mouse      GATTTT----AATTAGAAAA
Opossum    GATTTT----AATTAGAGAA
chick      GATTTT----AATTAGAGAA
Latimeria  GATTTT----AATTAGGGAG
Zebrafish  G-TTTTT---AATTAGAGca
Fugu       GGTTTTTT--AATTAAGGAt
Medaka     AGTTTTTTtaAATTAAGGAa
```
