# Supplementary material for: Conservation of shh cis-regulatory architecture of the coelacanth is consistent with its ancestral phylogenetic position
Source: EvoDevo. 2010 Nov 3;1:11. doi: 10.1186/2041-9139-1-11 (PMC2992049; doi:10.1186/2041-9139-1-11)
Supplement: Additional file 5 — Mapping of zebrafish ar-D. The experimental identification of the zebrafish ar-D enhancer is summarized. [file 2041-9139-1-11-S5.PDF]

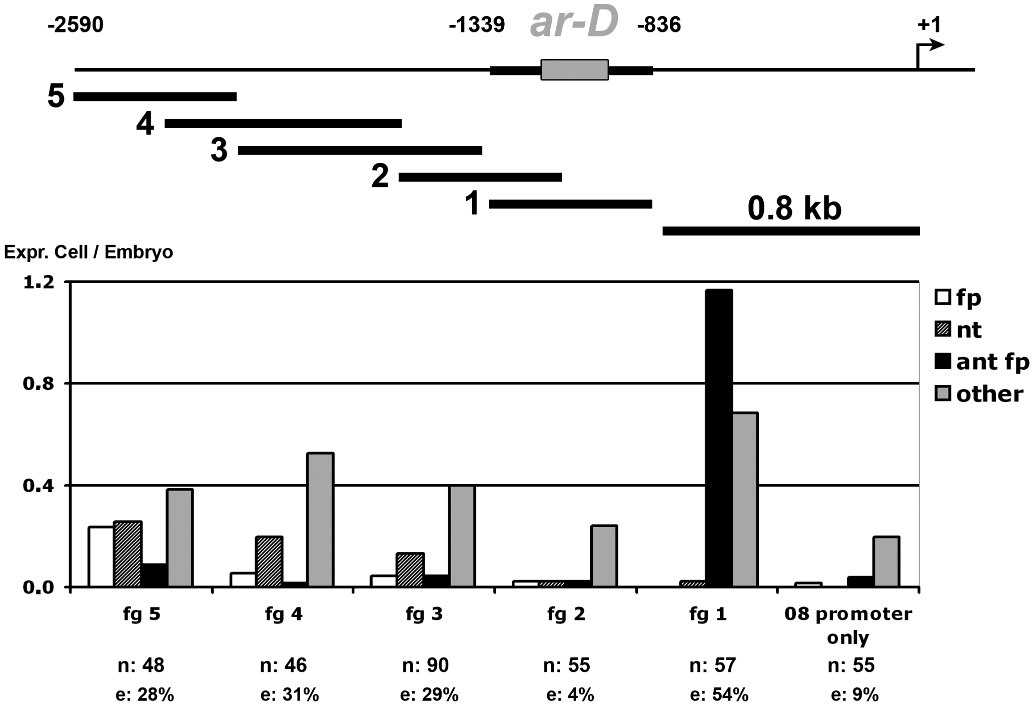

Mapping of zebrafish *arD*. Overlapping DNA fragments are indicated as black bars 1-5 that were employed in transient reporter gene expression analysis. Conservation to mouse SFPE1 enhancer is indicated grey. Numbers indicate the relative position of fragments within the 2.5 kb *shh* upstream region. The histogram shows ratios of LacZ expressing cells per embryo, quantified for each fragment in different tissues: neural tube (nt), floor plate (fp), anterior floor plate (ant fp) (anterior to yolk extension) and for randomly expressed cells (other). The number (n) of injected embryos and the percentage of expressing embryos (e) is indicated for each analysis.
