# Supplementary material for: Conservation of shh cis-regulatory architecture of the coelacanth is consistent with its ancestral phylogenetic position
Source: EvoDevo. 2010 Nov 3;1:11. doi: 10.1186/2041-9139-1-11 (PMC2992049; doi:10.1186/2041-9139-1-11)
Supplement: Additional file 6 — Oligonucleotides. The oligonucleotides for sub-cloning of enhancers and transgenic analysis are listed. [file 2041-9139-1-11-S6.PDF]

Oligonucleotides for Sub-cloning of enhancers and transgenic analysis.

Oligo-nucleotide	Sequence	Genomic region	Species
Fragment 1 F<br>Fragment 1 R	ATCATTTAATGGCATTTATA<br>GAGTGCGGTGTACAACTG	upstream -1339 / -836	*Danio rerio*
Fragment 2 F<br>Fragment 2 R	TGAGCCAGTTAATAGCAG<br>TTCCCTGCCCCATAGCAGCG	upstream -1590 / -1087	
Fragment 3 F<br>Fragment 3 R	TGCTATTTCTGAGCAGAATT<br>GACATGTTTTTGACCCATTTAG	upstream -2072 / -1340	
Fragment 4 F<br>Fragment 4 R	TACTGTGGAAACCTATGAAAAG<br>TTACTAAGCCCACACGGAATC	upstream -2329 / -1582	
Fragment 5 F<br>Fragment 5 R	TTTTTTTAGTGTAGCAGAAC<br>AAAAATGTCAGCAGGTTCTG	upstream -2590 / -2073	
LMarDcons<br>LMarDconsR	ATCTGTCGACGCATAGTGTCTGAACAGGAGTAA<br>ATCTCTGCAGCCAATGAGGTGTCAACAGAAA	*ar-D* (conserved)	*Latimeria menadoensis*
LMarDpl500F<br>LMarDpl500R	ATCTGTCGACGGTGGCGGAATCAACTTAGTAAA<br>ATTCCTGCAGAGGTGTTTCCAACGTGTTTATGA	*ar-D* (conserved + 500 bp up-/ downstream)	
LMarAconsF<br>LMarAconsR	ACTCGCGGCCGCAGGGAATTTAGAAATCATTGA<br>ACTCCCGCGGTCAAAGATTTTCCCTAGTACAG	*ar-A* (conserved)	
LMarFF<br>LMarFR	GACTGGCAAATTTCCTGTGACTTAG<br>AGGCTCTTTTTCTCCCAAAGACC	*CNE Intron 1, 588 bp fragment position 8088-8675*	
LMarGF<br>LMarGR	GAAAAGCTGCAAGACAGTGATCC<br>AGTGCAACCATCACATGAAAACC	*CNE Intron 1, 591 bp fragment position 9182-9773*	
LMarApl500F<br>LMarApl250R	ATCTGCGGCCGCTACGTTAATCTGGAGCTAGTCA<br>ATCTCCGCGGTTGAAGAAATAGCTTATGTTAGTT	*ar-A* (conserved + 500 bp up- + 250 bp downstream)	
LMarBconsF<br>LMarBconsR	ACTCGCGGCCGCTTTCAAGAATGGTGCTATGTAT<br>ACTCGGTACCAAATAATTTTAGCTTGTGCAATG	*ar-B* (conserved)	
LMarBpl250F<br>LMarBpl500R	ACTCGCGGCCGCAACTAACATAAGCTATTTCTTCAA<br>ATCTGGTACCGGTCTGTTTATTTCATGTTCAT	*ar-B* (conserved + 250 bp up- + 500 bp downstream)	
